# Cabergoline as a Novel Strategy for Post-Pregnancy Breast Cancer Prevention in Mice and Human

**DOI:** 10.21203/rs.3.rs-3854490/v1

**Published:** 2024-02-05

**Authors:** Natalia García-Sancha, Roberto Corchado-Cobos, Adrián Blanco-Gómez, Oriol Cunillera Puértolas, Mercè Marzo-Castillejo, Sonia Castillo-Lluva, Diego Alonso-López, Javier De Las Rivas, Julio Pozo, Alberto Orfao, Luis Valero-Juan, Carmen Patino-Alonso, David Perera, Ashok R. Venkitaraman, Jian-Hua Mao, Hang Chang, Marina Mendiburu-Eliçabe, Patricia González-García, Eduardo Caleiras, Isabel Peset, María Begoña García Cenador, Francisco Javier García-Criado, Jesús Pérez-Losada

**Affiliations:** Universidad de Salamanca-CSIC. IBSAL; Universidad de Salamanca-CSIC. IBSAL; Universidad de Salamanca-CSIC. IBSAL; Unitat de Suport a la Recerca Metropolitana Sud, Fundació Institut Universitari per a la recerca a l’Atenció Primària de Salut Jordi Gol i Gurina (IDIAPJGol), L’Hospitalet de LL; Unitat de Suport a la Recerca - IDIAP Jordi Gol. Direcció d’Atenció Primària Costa de Ponent, Institut Català de la Salut; Universidad Complutense Madrid; Cancer Research Center (CIC-IBMCC, CSIC/USAL), Consejo Superior de Investigaciones Científicas (CSIC) and University of Salamanca (USAL); Cancer Research Center (IBMCC, CSIC/USAL), Consejo Superior de Investigaciones Cientificas & University of Salamanca; Servicio de Citometría, Departamento de Medicina, Biomedical Research Networking Centre on Cancer CIBER-CIBERONC (CB16/12/00400), Institute of Health Carlos III, and Instituto de Biolog; University of Salamanca; Departamento de Ciencias Biomédicas y del Diagnóstico. Universidad de Salamanca.; Universidad de Salamanca. IBSAL.; The Medical Research Council Cancer Unit, University of Cambridge; Cancer Science Institute of Singapore; Lawrence Berkeley National Laboratory; Lawrence Berkeley National Lab; Instituto de Biología Molecular y Celular del Cáncer (IBMCC-CIC); Centro Nacional de Investigaciones Oncológicas (CNIO); Spanish National Cancer Research Centre; Spanish National Cancer Research Centre (CNIO), Madrid; Departamento de Cirugía, Universidad de Salamanca; Department of Surgery. University of Salamanca; Universidad de Salamanca-CSIC. IBSAL

**Keywords:** Cabergoline, Breast Cancer Prevention, Post-Pregnancy Breast Cancer, Brca1/P53-Deficient Mice, Postlactational Involution, Chemoprevention

## Abstract

Post-pregnancy breast cancer often carries a poor prognosis, posing a major clinical challenge. The increasing trend of later-life pregnancies exacerbates this risk, highlighting the need for effective chemoprevention strategies. Current options, limited to selective estrogen receptor modulators, aromatase inhibitors, or surgical procedures, offer limited efficacy and considerable side effects. Here, we report that cabergoline, a dopaminergic agonist, reduces the risk of breast cancer post-pregnancy in a *Brca1/P53*-deficient mouse model, with implications for human breast cancer prevention. We show that a single dose of cabergoline administered post-pregnancy significantly delayed the onset and reduced the incidence of breast cancer in *Brca1/P53*-deficient mice. Histological analysis revealed a notable acceleration in post-lactational involution over the short term, characterized by increased apoptosis and altered gene expression related to ion transport. Over the long term, histological changes in the mammary gland included a reduction in the ductal component, decreased epithelial proliferation, and a lower presence of recombinant *Brca1/P53* target cells, which are precursors of tumors. These changes serve as indicators of reduced breast cancer susceptibility. Additionally, RNA sequencing identified gene expression alterations associated with decreased proliferation and mammary gland branching. Our findings highlight a mechanism wherein cabergoline enhances the protective effect of pregnancy against breast cancer by potentiating postlactational involution. Notably, a retrospective cohort study in women demonstrated a markedly lower incidence of post-pregnancy breast cancer in those treated with cabergoline compared to a control group. Our work underscores the importance of enhancing postlactational involution as a strategy for breast cancer prevention, and identifies cabergoline as a promising, low-risk option in breast cancer chemoprevention. This strategy has the potential to revolutionize breast cancer prevention approaches, particularly for women at increased risk due to genetic factors or delayed childbirth, and has wider implications beyond hereditary breast cancer cases.

Breast cancer stands as the foremost cancer in women globally, leading in cancer-related deaths (1). This prevalence is influenced by factors such as increased life expectancy and a trend towards pregnancies after age 30, factors that have been implicated in the elevated incidence rates in Western societies (2–4). For instance, the United States has observed an annual increase in incidence rates by approximately 0.5% (5).

A critical epidemiological aspect is the heightened risk of breast cancer shortly after pregnancy, lasting up to a decade, especially in women who bear children after age 35 (4, 6). This risk is underscored by a demographic shift towards later maternal age. In the U.S., the proportion of births to women over 35 increased from 8% in 1990 to 18% in 2018 (7). Alarmingly, an increasing incidence of breast cancer is noted among younger women (15 to 44 years), with post-pregnancy breast cancer accounting for a substantial portion of these cases. Notably, this cancer subtype, regardless of estrogen receptor (ER) status, is often marked by a more aggressive course, with increased risks of metastasis and mortality(8).

Current chemopreventive strategies, such as selective estrogen receptor modulators (SERMs) and aromatase inhibitors (AIs), are hampered by adverse effects including endometrial cancer and thrombosis (9). Surgical options like mastectomy and oophorectomy, though effective in drastically reducing risk, particularly in hereditary breast cancer, are associated with significant physical and psychological repercussions (10–12). Chemoprevention is typically reserved for women at very high risk (Gail score >3%), yet its utilization is impeded by side effects, leading to a 40% discontinuation rate in the prescribed 5-year regimen (13, 14). Moreover, these interventions offer temporary efficacy and do not entirely eliminate risk, as evidenced by the modest reductions in invasive breast cancer incidences achieved by tamoxifen and raloxifene (9). Critically, there are no specific preventive strategies for post-pregnancy and hormone receptor-negative breast cancers, frequently observed in *BRCA1* mutation carriers (15).

Early childbearing, particularly before the age of 30, significantly reduces the long-term risk of breast cancer. This protective effect is inversely proportional to maternal age at first childbirth, with earlier ages conferring greater benefits (16). Early pregnancy offers partial protection against both ER-positive and ER-negative breast cancers in women with *BRCA1* mutations (17–19), with each subsequent pregnancy further enhancing this protective effect (20).

The protective influence of pregnancy against breast cancer is a universal phenomenon, observed across diverse ethnic groups and in mammalian models (21–23). Various cellular subpopulations and signaling pathways are implicated in this protection (24–26), yet the precise mechanisms remain elusive.

During pregnancy, the mammary gland’s epithelial proliferation prepares for milk production. Post-lactation, the gland undergoes involution, marked by cell death and structural reversion, influenced by hormonal changes involving dopamine and prolactin (27). Delayed involution may encourage tumorigenesis, while complete involution could be protective. Indeed, studies in genetically modified models show that delayed involution increases, while accelerated involution decreases, breast cancer risk. For instance, in *Snai2* or *Bax* knockouts and *Bcl2* or *Akt1* overexpressing mice, delayed involution results in higher epithelial tissue retention, potentially harboring malignant cells (28–35). Conversely, *Akt1*-deficiency and *Sta5a*-deficiency in mice lead to early involution and reduced cancer risk (36–39). This suggests postlactational involution as a potential mechanism to eliminate DNA-mutated epithelial cells, reducing long-term breast cancer risk.

Our study proposes that enhancing postlactational involution could lower breast cancer risk by eliminating damaged epithelial cells more effectively. We explored the use of cabergoline, a dopaminergic agonist known for prolactin inhibition, previously applied in dairy cows for faster involution (40–42) and to suppress lactation in non-breastfeeding women (43, 44).

We demonstrate that cabergoline, by intensifying postlactational involution in mice, amplifies the pregnancy’s protective effect against breast cancer. This effect involves histological and molecular alterations in the mammary glands, reducing their susceptibility to malignancy. Our findings also show that cabergoline may decrease the risk of post-pregnancy breast cancer in women.

## RESULTS

### Enhanced breast cancer protection in *Brca1/P53*-deficient mice post-cabergoline treatment in pregnancy context

The well-established protective effect of pregnancy against breast cancer, documented across diverse mammalian models including mice and rats (16, 21–23), was further explored in this study using the *Brca1/P53*-deficient mouse model. These mice, predisposed to developing triple-negative basal breast cancer (45), were utilized to determine if pregnancy’s protective effects could be potentiated in a genetically susceptible context.

**Figure 1:**
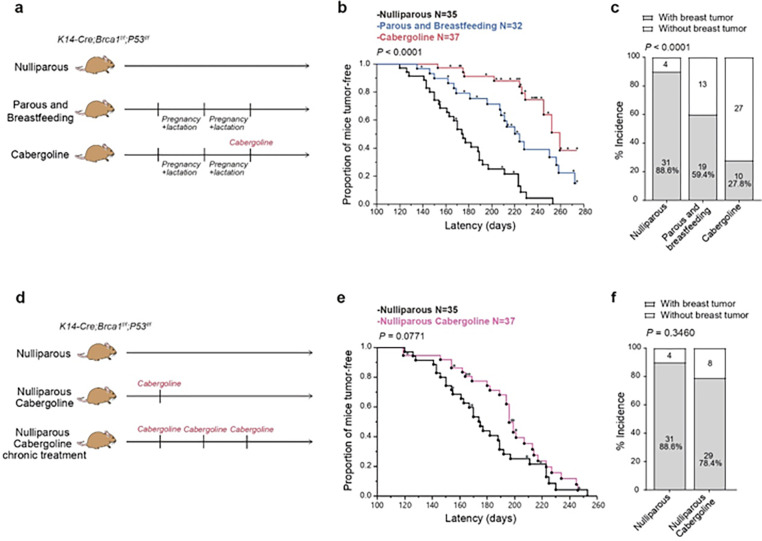
Impact of cabergoline on breast cancer risk in Brca1/P53-deficient mice post-pregnancy. **a**) Study design for three cohorts: nulliparous mice, multiparous mice having completed two pregnancies with lactation, and a third group of multiparous mice treated with cabergoline post-lactation. **b, c**) Tumor latency (b) and incidence (c) comparisons between nulliparous and multiparous mice, with and without cabergoline treatment. **d**) Additional experimental groups: nulliparous mice receiving a single cabergoline dose, and another receiving biweekly doses. **e, f**) Analysis of tumor latency (e) and incidence (f) in nulliparous mice treated with cabergoline. Tumor latency and incidence assessed using Kaplan-Meier survival curves (a, b) and Chi-square tests (c, f), respectively. Further exploration of cabergoline’s potential protective effect in nulliparous *Brca1/P53*-deficient mice revealed a marginal and statistically non-significant decrease in tumor latency (P = 0.0771), with no observable change in tumor incidence (P = 0.3460) (Fig. 1d, f). A biweekly cabergoline regimen in a separate nulliparous cohort showed no significant alterations in tumor incidence or latency (**Extended Data Fig. 1g, h**), indicating the absence of a dose-dependent protective effect of cabergoline in the context of non-pregnancy.

In this comprehensive longitudinal study, female *Brca1/P53*-deficient mice underwent two full reproductive cycles, including pregnancy and lactation. For the purposes of this manuscript, these mice will be referred to interchangeably as either ‘parous and breastfeeding mice’ or ‘multiparous mice’ (Fig. 1a). This cohort was compared to nulliparous counterparts to assess the protective effect of pregnancy. The results indicated a significant delay in breast cancer onset in multiparous mice (median onset shifted from 175 to 224 days; P = 0.0002) and a notable reduction in tumor incidence (from 88.6% to 59.4%; P = 0.0061) (Fig. 1b, c and **Extended Data Fig. 1a, b**).

Given the known relationship between postlactational involution and breast cancer risk in several genetically modified mouse models, where delayed involution is associated with increased risk and accelerated involution with decreased risk (27), we explored the impact of cabergoline, a drug recognized for hastening postlactational involution in dairy cows (41, 42). Our focus was on its efficacy in altering breast cancer development post-pregnancy.

Administering a single dose of cabergoline at the conclusion of the second lactation period resulted in a marked extension of tumor latency in multiparous mice, compared to both untreated multiparous and nulliparous groups (extension to 259 days; P = 0.0119 and P < 0.0001, respectively) (Fig. 1b and **Extended Data Fig. 1c, d**). Moreover, cabergoline treatment notably decreased tumor incidence in multiparous mice, with a reduction to 27.8%, in contrast to 88.6% in nulliparous and 59.4% in untreated multiparous counterparts. These differences were statistically significant (P < 0.0001 and P < 0.0065, respectively) (Fig. 1c and **Extended Data Fig. 1e, f**).

Further exploration of cabergoline’s potential protective effect in nulliparous *Brca1/P53*-deficient mice revealed a marginal and statistically non-significant decrease in tumor latency (P = 0.0771), with no observable change in tumor incidence (P = 0.3460) (Fig. 1d, f). A biweekly cabergoline regimen in a separate nulliparous cohort showed no significant alterations in tumor incidence or latency (**Extended Data Fig. 1g, h**), indicating the absence of a dose-dependent protective effect of cabergoline in the context of non-pregnancy.

These findings collectively underscore the significance of post-pregnancy cabergoline treatment in substantially reducing breast cancer risk in a genetically susceptible model, an effect not replicated in the absence of pregnancy.

### Cabergoline’s role in enhancing postlactational involution in *Brca1/P53*-deficient mice

Building on the premise that accelerated postlactational involution is inversely correlated with breast cancer risk (27), this study delved into the mechanisms by which cabergoline augments pregnancy-related breast cancer protection. A key focus was to determine if cabergoline indeed amplifies postlactational involution in a *Brca1/P53*-deficient mouse model.

**Figure 2:**
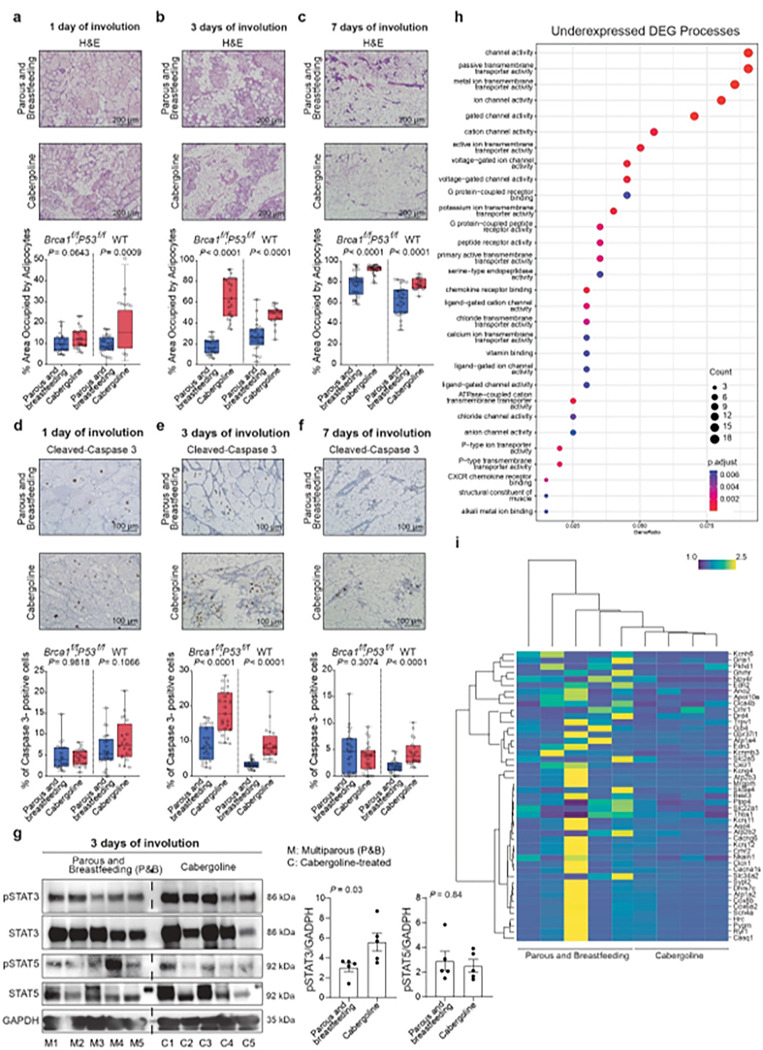
Cabergoline-induced changes in mammary glands of Brca1/P53-deficient Mice. **a-c**) Histopathological examination of mammary gland adipocyte expansion at 1, 3, and 7 days post-lactation, comparing cabergoline-treated and untreated mice. **d-f**) Apoptosis analysis using cleaved-caspase 3 staining at corresponding timepoints; a-f, Five mice per group were evaluated, encompassing both Brca1/P53-deficient and wild-type cohorts. **g**) Assessment of pSTAT3 and pSTAT5 protein levels in mammary gland organoids on day 3 post-treatment of five mice per group. a-g, Mann-Whitney U test. **h**) Gene Ontology (GO) analysis highlighting downregulated genes related to ion homeostasis. **i**) Heatmap illustrating the differential expression of ion transporter genes post-cabergoline treatment. Mammary glands from five multiparous Brca1/P53-deficient mice, both untreated and four treated with cabergoline, were analyzed 24 hours post-cabergoline administration.

In our detailed investigations, mammary glands of cabergoline-treated *Brca1/P53*-deficient mice were examined at intervals post-lactation. The findings revealed an accelerated involution process, characterized by an expanded adipocyte area at three and seven days post-treatment (P < 0.0001), suggesting an early onset of involution (Fig. 2a-c). A corresponding increase in apoptotic epithelial cells at the 3-day mark (P < 0.0001) further confirmed this observation (Fig. 2d-f). These trends were also evident in wild-type mice, reinforcing the role of cabergoline in involution acceleration (Fig. 2a-f).

We then explored the molecular underpinnings of this accelerated involution. The focus was on pSTAT3 and pSTAT5, molecules pivotal in initiating postlactational involution (38, 46). In cabergoline-treated mice, an upregulation of pSTAT3 was observed (Fig. 2g), aligning with the drug’s involution-enhancing effects. Subsequent RNA-seq analysis of mammary glands 24 hours post-lactation identified 14 differentially expressed genes (DEGs) (P Adj ≤ 0.05) between untreated and cabergoline-treated groups, although initial enrichment analysis did not directly link these changes to involution (**Supplementary Tables S1, S2**).

To elucidate the underlying mechanisms of our observed increase in cell death during post-lactation involution, we refined our analytical approach. Initially, we identified differentially expressed genes (DEGs) using a threshold of P ≤ 0.05. Subsequent enrichment analysis of these DEGs enabled us to focus on specific biological processes relevant to our study. This approach, in line with established methodologies (47–51), enabled the identification of 263 overexpressed and 347 underexpressed transcripts in treated mice, facilitating distinct clustering of mammary gland samples from treated and untreated mice (**Extended Data Fig. S2a** and **Supplementary Table S3**).

Enrichment analysis pinpointed underexpressed genes in cabergoline-treated mice associated with homeostasis and ion transport, alongside overexpressed genes linked to immune processes (Fig. 2h, i, **Extended Data Fig. 2b**, and **Supplementary Table S4**). Ion channel homeostasis, particularly involving calcium ions, plays a crucial role in apoptosis (52–58). The observed changes in specific ion transporters in cabergoline-treated mice are consistent with the initiation of involution, potentially through elevated intracellular calcium levels (53–58). These patterns were consistent across various segments of the analysis (**Supplementary Table S5**).

The data collectively indicate that cabergoline enhances post-lactational involution, marked by increased apoptosis and accelerated adipocyte expansion (Fig. 2a-f). The drug appears to facilitate involution by regulating ion channel homeostasis, particularly impacting calcium ion transport, thus enhancing cell death and promoting involution.

### Long-term impact of cabergoline on mammary gland ductal structure and proliferation dynamics in *Brca1/P53*-deficient mice

Given the established correlation between mammographic breast density and breast cancer risk (59, 60), and the inverse relationship between glandular fraction and mammary involution (61), we focused on the long-term effects of cabergoline on mammary gland composition as an indirect measure of breast cancer susceptibility (62, 63).

**Figure 3:**
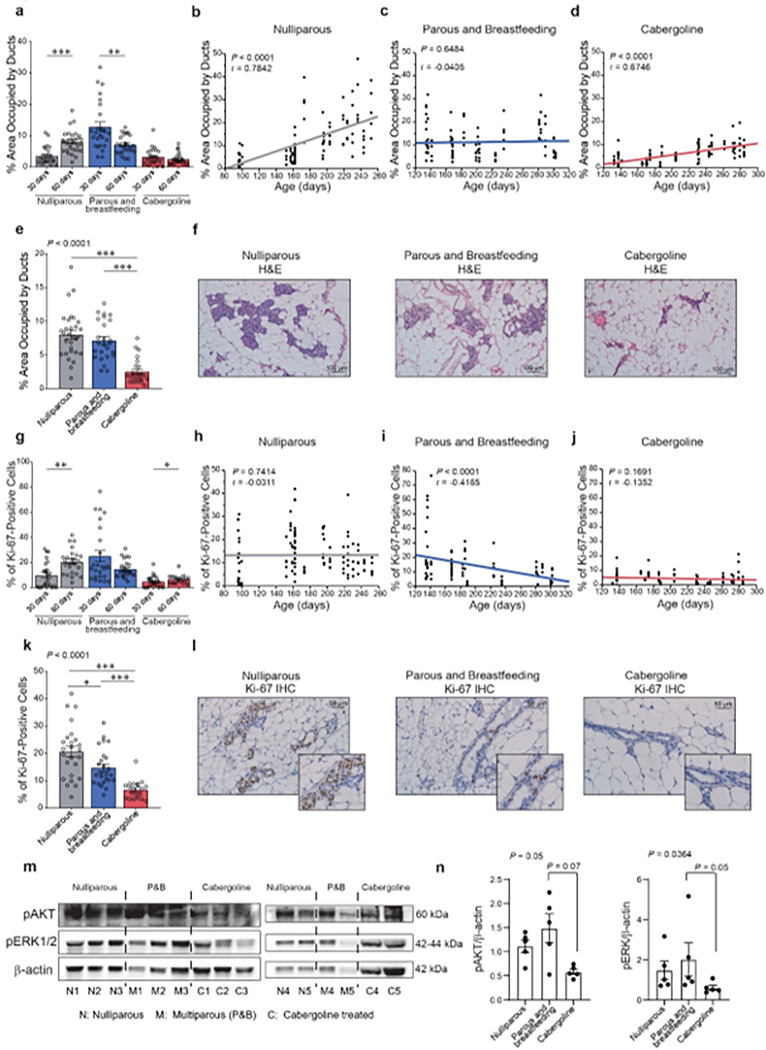
Extended effects of cabergoline on mammary gland morphology and proliferation in Brca1/P53-deficient mice. **a**) Ductal area assessment at 30 and 60 days post-lactation in nulliparous and multiparous mice, untreated and treated with cabergoline (five mice per group). **b-d**) Temporal changes in ductal area across nulliparous (N = 23), multiparous (N = 21), and cabergoline-treated (N = 23) mice. **e, f**) Analysis of ductal area at 60 days post-lactation, including histological images. **g**) Proliferation evaluation via Ki-67 staining at 30 and 60 days post-lactation (five mice per group). **h-j**) Longitudinal patterns of ductal proliferation across different groups: nulliparous (N = 23), multiparous (N = 21), and cabergoline-treated (N = 21) mice. **K, I**) Detailed proliferation analysis at 60 days post-lactation. **m, n**) Analysis of pAKT and pERK protein levels, quantified using Western blot and ImageJ software (five mice per group). Panels a, e, g, k, n used the Kruskal-Wallis test and Wilcoxon post-test. Panels b-d and h-j applied the Spearman test. Statistical significance indicated as *** P<0.0005; ** P<0.005; * P<0.05.

This study employed a cross-sectional and longitudinal approach to assess the glandular tissue evolution in *Brca1/P53*-deficient mice post-cabergoline treatment (Fig. 3).

In the cross-sectional analysis, we quantified ductal tissue in *Brca1/P53*-deficient mice post-involution. The comparison involved multiparous mice, both untreated and treated with cabergoline, at 30- and 60-days post-involution, alongside nulliparous, age-matched controls. Nulliparous mice exhibited an increased glandular component over time (P < 0.0001), while multiparous mice showed a decrease (P = 0.0038). Notably, cabergoline-treated mice consistently demonstrated the least glandular component, with minimal variation between the two time points (Fig. 3a).

In the longitudinal analysis, which included mice previously assessed for breast cancer susceptibility (Fig. 1), we observed a progressive increase in ductal area in nulliparous mice (P < 0.0001; r = 0.7842) (Fig. 3b). This pattern was not evident in multiparous mice (Fig. 3c). Intriguingly, cabergoline-treated mice maintained a smaller initial ductal area that increased minimally with age (Fig. 3d), but consistently less than in untreated mice. The minimal ductal area in the cabergoline-treated group was confirmed at 60 days post-involution (Fig. 3e, f), aligning with our hypothesis of cabergoline’s role in reducing breast cancer susceptibility. Comparing cabergoline’s effect on multiparous wild-type mice, we observed a consistent reduction in ductal area (**Extended Data Fig. 3a-c**).

To further explore the impact of cabergoline on mammary gland dynamics, we assessed apoptosis and epithelial proliferation in *Brca1/P53*-deficient mice. The cabergoline-treated group showed a significantly higher apoptotic rate than untreated multiparous mice (P = 0.0268) (**Extended Data Fig. 3d, e**), albeit minimal across all groups. Ki-67 expression, a marker for basal proliferation and a recognized breast cancer risk factor (64–66), was employed to quantify glandular tissue proliferation. Thus, we used this marker to assess glandular tissue proliferation as an indicator of breast cancer susceptibility. We found a significant increase in epithelial cell proliferation from 30 to 60 days in nulliparous mice (P = 0.0006), a trend absent in multiparous mice. Cabergoline-treated mice exhibited markedly lower proliferation rates at both time points (Fig. 3g).

Our longitudinal study further demonstrated that in nulliparous mice, the proliferation rate of cells in the mammary gland did not exhibit significant temporal variations (P = 0.74144; r = −0.0311) (Fig. 3h). However, this rate significantly decreased post-pregnancy (P < 0.0001; r = −0.4165) (Fig. 3i). The cabergoline-treated group consistently exhibited the lowest proportion of proliferating cells throughout their lifespan (P = 0.1691; r = −0.1352) (Fig. 3j), with the most pronounced decrease in epithelial cell proliferation confirmed at 60 days post-involution (Fig. 3k, l).

Comparisons in wild-type mice at 30 and 60 days post-involution, both treated and untreated with cabergoline, corroborated these findings (**Extended Data Fig. 3f-h**). Notably, the proliferation rate in nulliparous wild-type mice was lower than in their *Brca1/P53*-deficient counterparts (**Extended Data Fig. 3i**), emphasizing the potential role of basal breast tissue proliferation in breast cancer susceptibility (64–68).

Lastly, we linked the diminished proliferation in cabergoline-treated *Brca1/P53*-deficient mice to reduced pAKT and pERK levels in breast organoids, as determined by Western blot analysis (Fig. 3m, n).

These findings suggest that extended pregnancy, followed by cabergoline treatment, leads to a reduction in glandular components and suppresses ductal proliferation, thereby aligning with the reduced breast cancer susceptibility observed in *Brca1/P53*-deficient mice (Fig. 1b, c and **Extended Data Fig. 1a-f**).

### Deciphering the molecular mechanisms underlying decreased breast cancer risk in multiparous *Brca1/P53*-deficient mice through RNA-seq analysis

In an effort to unravel the molecular basis for the observed reduction in breast cancer risk in multiparous *Brca1/P53*-deficient mice, we carried out a comprehensive RNA-seq study. We analyzed non-tumoral breast tissues from nulliparous and multiparous mice 60 days post-lactational involution. This comparative approach identified distinct sets of differentially expressed genes (DEGs) (P Adj ≤ 0.05) between these groups, providing insights into the molecular divergence contributing to the altered cancer susceptibility (**Extended Data Fig. 4a** and **Supplementary Table S6**).

Utilizing Gene Ontology (GO) enrichment, Reactome, and WikiPathways analyses, we categorized these DEGs into functionally relevant biological processes and pathways. The enrichment analysis highlighted a spectrum of biological processes and pathways associated with both overexpressed and underexpressed genes in the multiparous group compared to their nulliparous counterparts (**Supplementary Table S7**). Key biological processes are depicted in **Extended Data Fig. 4b, c**.

**Figure 4:**
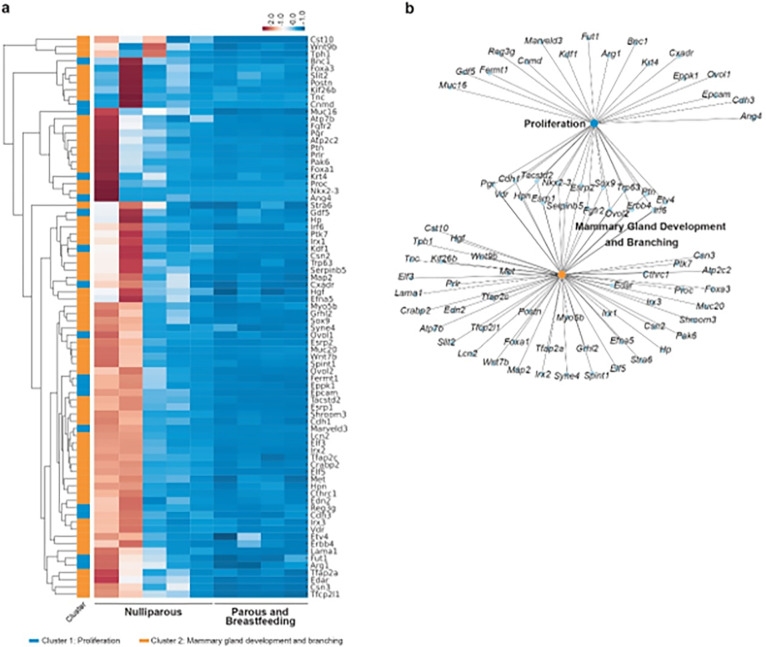
Transcriptomic profiling in Brca1/P53-deficient mice reflecting the impact of parity on mammary gland biology. **a**) Heatmap analysis: visual comparison of gene expression between nulliparous (N = 5) and multiparous mice (N = 4). This heatmap focuses on genes linked to mammary gland development, proliferation, and branching, highlighting those significantly underexpressed in multiparous mice. **b**) Network visualization: illustrates the interconnected relationships of highlighted genes using Kamada-Kawai layout, emphasizing their roles in mammary gland biology, including proliferation, development, and branching.

Correlating our findings with the progressive increase in ductal area over time in nulliparous mice (Fig. 3b, c), and the attenuated proliferation observed in multiparous mice (Fig. 3h, i, k), we focused on clusters related to tubular growth, breast development, and cellular proliferation (Fig 4a and **Supplementary Tables S8, S9**). Pregnancy consistently resulted in lower gene expression within these clusters in multiparous mice. In contrast, nulliparous mice showed a higher and broader range of transcriptomic variability in non-tumoral mammary glands (Fig. 4a, b), aligning with the observed differences in ductal area and proliferation rates.

The underexpression of genes associated with cell proliferation, mammary gland development, and branching in multiparous mice’s breasts may be a key factor in their reduced cancer susceptibility. Critical genes in this context include *Pgr, Sox9, Fgfr2, Erbb4*, and the *Hgf/Met* signaling axis, among others (**Extended Data Fig. 4d**). The altered expression of *Pgr* suggests shifts in hormonal signaling pivotal to mammary tissue development and proliferation (69, 70). *Sox9*, essential in stem cell differentiation (71), alongside *Fgfr2* and *Erbb4*, crucial for cell survival and signaling (72–77), indicate a protective shift against cancer cell proliferation and transformation. The role of the *Hgf/Met* pathway in cell proliferation and mammary morphogenesis further underscores its potential contribution to mitigating cancer initiation and progression (78, 79).

In summary, these molecular patterns suggest a link between diminished activity in specific pathways and the reduced glandular development and proliferation observed in multiparous mice. This molecular landscape potentially underlies the lowered breast cancer susceptibility in this specific mouse model, providing valuable insights into the interplay between genetic factors, pregnancy, and cancer risk.

### RNA-Seq analysis of cabergoline-induced gene expression changes in mammary glands of multiparous mice

Later, we expanded our investigation to elucidate the impact of cabergoline treatment on gene expression in the mammary glands of multiparous mice. Through comparative RNA-Seq analysis, distinct differentially expressed genes (DEGs) (P Adj ≤ 0.05) were identified between cabergoline-treated and untreated groups, as detailed in **Supplementary Table S10**. Heatmap analyses effectively demarcated the gene expression profiles of these cohorts (**Extended Data Fig. 5a**).

Utilizing Gene Ontology (GO), Reactome, and Wikipathways analyses, we categorized these DEGs into significant biological pathways impacted by cabergoline treatment in multiparous mice. These pathways, crucially altered by cabergoline, might elucidate the molecular underpinnings of its role in augmenting breast cancer protection post-pregnancy (**Supplementary Table S11**). Noteworthy among these pathways are those associated with attenuated inflammatory responses and modulated TNFα and insulin/IGF1 sensitivity (**Supplementary Table S12**), key elements in breast cancer susceptibility (80, 81).

**Figure 5:**
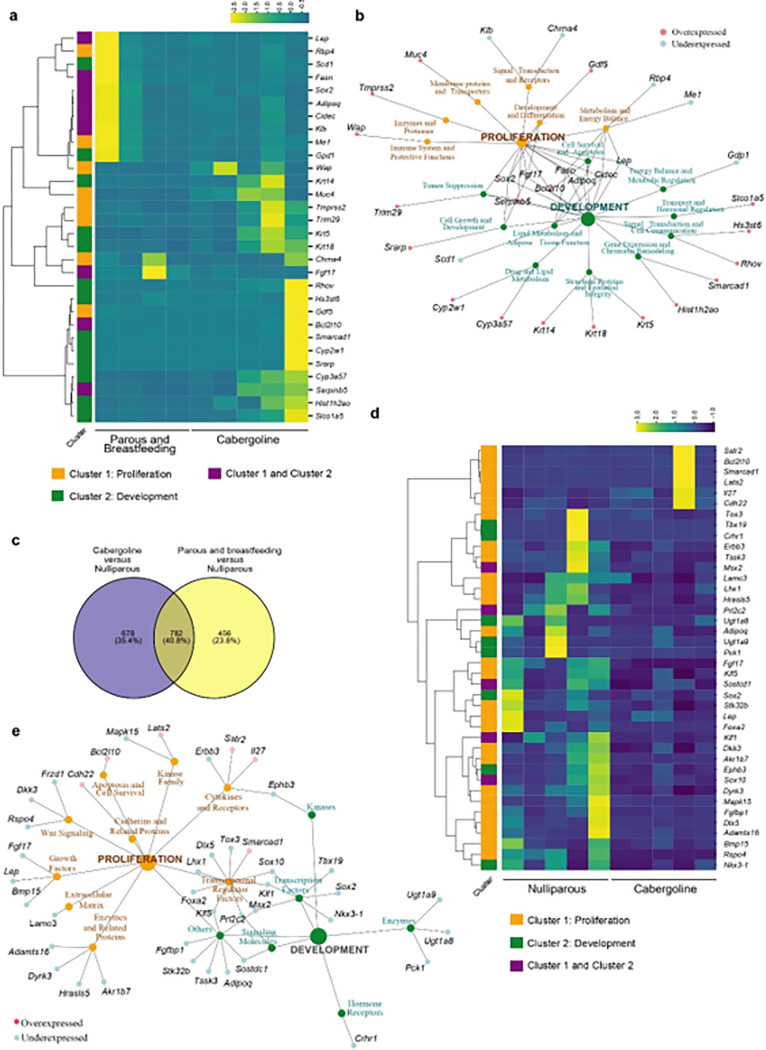
Gene expression dynamics in cabergoline-treated Brca1/P53-deficient mice. **a**) Heatmap analysis: compares gene expression in cabergoline-treated (N = 5) versus untreated (N = 4) multiparous mice’s mammary glands. Focuses on genes associated with proliferation and development. **b**) Gene network visualization: maps differentially expressed genes linked to proliferation and development, highlighting underexpressed and overexpressed genes in cabergoline-treated mice using the Kamada-Kawai layout. **c**) Venn diagram analysis: depicts the overlap and unique DEGs between multiparous (treated and untreated) and nulliparous mice, focusing on cabergoline-specific gene expression changes (purple section). **d, e**) Targeted gene expression analysis: heatmap and network analysis of genes uniquely altered in cabergoline-treated mice, emphasizing their potential roles in reducing breast cancer risk through altered mammary gland development and proliferation.

Following the observation of a reduced glandular component and lower proliferation rates in *Brca1/P53*-deficient mice’s mammary glands post-cabergoline treatment (Fig. 3), we honed in on pertinent DEGs related to proliferation and development (Fig. 5a, b; **Supplementary Tables S12, S13**). Among others, cabergoline was found to upregulate genes such as *Krt14, Serpinb5*, and *Trim29* (**Extended Data Fig. 5b**), potentially augmenting cellular differentiation, DNA stability, and immune responses. Of particular interest, *Trim29* may offer protective roles against DNA damage and inflammation-associated cancer, impacting estrogen receptor (ER) signaling (82, 83), while *Serpinb5 (Maspin)* is implicated in tumor suppression in mammary cells (84). Decreased expression of genes like *Scd1, Fasn, Sox2*, and *Lep* suggests a reduction in fat production and pluripotency (85, 86); additionally, elevated Leptin levels have been linked to mammary gland hyperplasia (87) (**Extended Data Fig. 5b**). These genetic alterations might underpin the observed reduced proliferation and diminished breast cancer risk in cabergoline-treated mice.

To further dissect cabergoline’s specific effects within the context of parity, we compared gene expression profiles between untreated and cabergoline-treated multiparous mice with those of nulliparous mice. DEGs (P Adj ≤ 0.05) unique to cabergoline-treated multiparous mice were examined (Fig. 5c; **Extended Data Fig. 5c; Supplementary Table S14**), revealing pathways that might underlie cabergoline’s enhanced protective effect against breast cancer, particularly those involving underexpressed DEGs related to inflammation and glandular development (**Supplementary Table S15**).

Our focus extended to genes implicated in proliferation and development, informed by literature and our enrichment study (Fig. 5d, e; **Supplementary Table S16**). In cabergoline-treated multiparous mice, key genes such as *ErbB3, Klf5, Igfbp1, Fgf17, Msx2*, and *Sox10* (88–92), among others, were notably underexpressed compared to nulliparous counterparts (**Extended Data Fig. 5d**). This underexpression suggests a plausible mechanism for the observed reduction in breast cancer risk, potentially due to constrained mammary gland growth and regulated cell proliferation, particularly in these pivotal genes governing mammary gland development and cell signaling (88–92).

In sum, these results provide a detailed molecular landscape that might elucidate how cabergoline administration in multiparous mice might confer a reduced risk of breast cancer.

### Influence of cabergoline on reducing *Brca1/P53*-deficient cell populations in mammary glands

Informed by the concept of “field cancerization,” which links an increased number of pre-malignant cells in a tissue to heightened cancer risk (93, 94), our study examined the long-term impact of cabergoline on the prevalence of *Brca1/P53*-deficient cells in the mammary gland. This analysis is pivotal given that in *Brca1/P53*-deficient mice, these cells, generated through Cre-recombinase action on K14+ cells, are the potential tumor progenitors (45). We sought to ascertain whether pregnancy and cabergoline treatment would alter the ratio of these recombinant cells, using this as a barometer for breast cancer risk.

**Figure 6:**
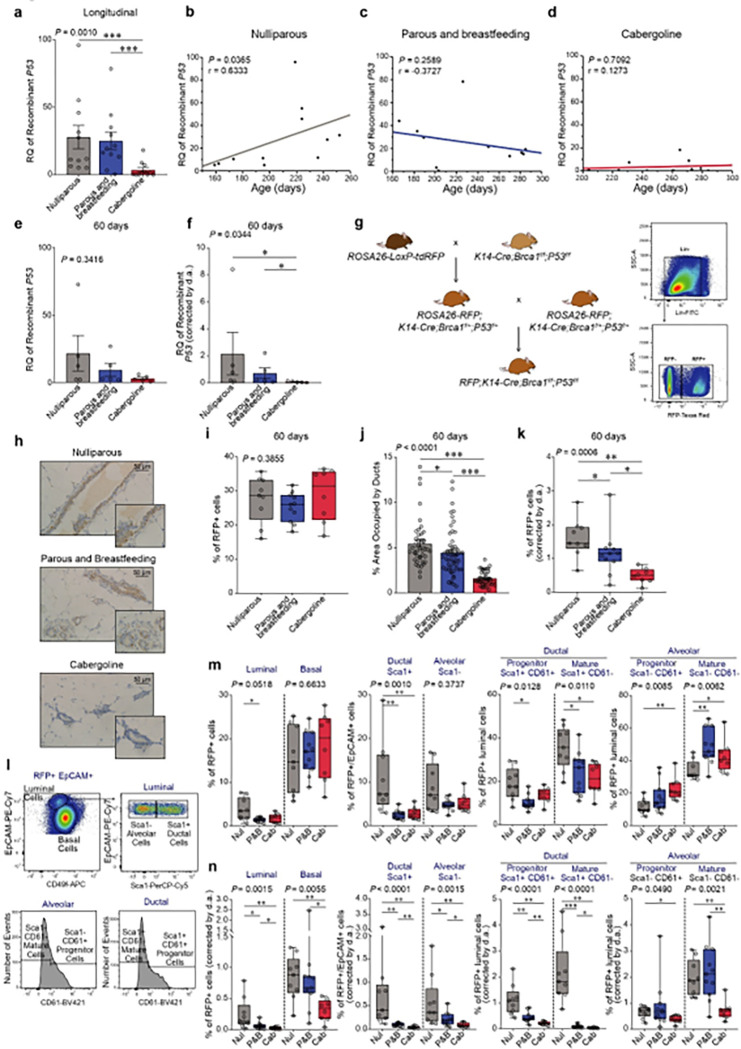
Analysis of recombinant cell dynamics and epithelial subpopulations in Brca1/P53-deficient mice. **a**) Quantitative PCR analysis: measures the recombinant P53 allele in various mouse groups of the longitudinal cohort to assess the impact of cabergoline treatment. **b-d**) Correlation studies: investigate the relationship between recombinant P53 allele quantity and age of mice in different cohorts; a-d, 11 mice per group were evaluated. **e**) Comparative analysis: cross-sectional comparison of the P53 allele across mouse groups. **f**) Recombinant cell quantification: adjusts the amount of recombinant cells for ductal area differences; e, f, five mice per group were assessed. **g**) RFP+ recombinant mouse model: describes the methodology for generating RFP+ recombinant Brca1/P53-deficient mice. **h**) RFP detection: immunohistochemical analysis of RFP-positive cells in mammary glands of various groups. **i**) Flow cytometry analysis: quantifies RFP+ cells in Brca1/P53-deficient mice. **j**) Ductal area impact: examines the effect of cabergoline on ductal area in RFP+ mice. **k**) Absolute RFP+ cell count: adjusts the number of RFP+ cells for ductal area (d.a.). **l**) Subpopulation assessments: flow cytometry study of the effect of cabergoline on recombinant breast epithelial subpopulations. **m, n**) Epithelial subpopulation examination: analyzes specific RFP+ epithelial subpopulations (m), with corrections for ductal area (d.a.) differences (n).i-n, nulliparous (N = 9), multiparous (N = 11), and cabergoline-treated (N =8) mice Statistical analysis includes Kruskal-Wallis test and Wilcoxon post-test (Panels a, e, f, i, j, k, m, n) and Spearman test (Panels b-d). Significance denoted as *** P < 0.0005; ** P < 0.005; * P < 0.05.

Quantifying the fraction of recombinant cells within the mammary gland of *Brca1/P53*-deficient mice, where 10–40% of cells are recombinant and predisposed to tumorigenesis (45), we utilized qPCR, an established method for cellular chimerism detection (95, 96). Our research reveals that the proportion of recombinant cells was significantly lower in cabergoline-treated multiparous mice compared to the other groups of mice in the longitudinal cohort (P < 0.001) (Fig. 6a).

An upward trend in recombinant cells deficient in Brca1/P53 was observed over time in nulliparous mice (P = 0.0365; r = 0.6333.) (Fig. 6b). However, this increase was not evident in either untreated or cabergoline-treated multiparous mice (P = 0.2589 and P = 0.7092, respectively) (Fig. 6c, d). At 60 days post-weaning, the cross-sectional cohort revealed no significant differences in the proportion of recombinant cells (P = 0.3416) (Fig. 6e), suggesting that the impact of cabergoline on recombinant cell populations becomes more pronounced over time. Notably, after adjusting for the cabergoline-induced reduction in ductal area, a substantial decrease in P53 recombinant alleles was evident in treated multiparous mice (P = 0.0344) (Fig. 6f).

Further validation was pursued using a second mouse model with Red Fluorescent Protein (RFP)+ *Brca1/P53*-deficient cells (Fig. 6g, h and **Supplementary Figure S1**)(97). Flow cytometry quantification of these cells post-lactational involution showed no significant differences at 60 days (P = 0.3855), but a marked reduction in ductal area was observed in cabergoline-treated mice (P < 0.0001) (Fig. 6i, j), accompanied by a decrease in recombinant RFP+ cells after adjusting for ductal area (P = 0.0006) (Fig. 6k).

Flow cytometry was also employed to dissect changes in various RFP+ recombinant epithelial cell subpopulations (Fig. 6l). Pregnancy and cabergoline treatment led to a reduced proportion of luminal cells (characterized by low CD49f and high EpCAM), with this effect pronounced in untreated multiparous mice compared to nulliparous counterparts (Fig. 6m, left). No significant difference was observed between cabergoline-treated and untreated multiparous groups across all cell subpopulations (Fig. 6m). Yet, accounting for the cabergoline-induced reduction in ductal area, all recombinant cell subpopulations exhibited a decline in the treated group (Fig. 6n).

Given the hypothesis that *BRCA1*-deficient breast cancer originates from luminal precursors (98, 99), the observed decrease in luminal cells implies that both pregnancy and cabergoline may diminish the pool of potential tumor-initiating cells. This effect was also reflected in RFP-negative cells, indicating a broader impact of the interventions (**Extended Data Fig. 6a, b**).

In summary, cabergoline treatment in parous mice significantly lowers the count of *Brca1/P53*-deficient cells, chiefly by reducing the glandular component. This consequential decrease narrows the ‘field cancerization’ zone, potentially enhancing post-pregnancy protective effects against breast cancer development.

### Assessment of cabergoline’s post-pregnancy protective effect against breast cancer in women

Our study embarked on a retrospective dynamic cohort analysis (2005–2021) to explore the potential protective impact of cabergoline administered post-pregnancy against breast cancer. Utilizing data from the Information System for the Development of Research in Primary Care (SIDIAP) database (100, 101) (**Extended Data Fig. 7**), we scrutinized the breast cancer incidence among women who had their first child at age 30 or older. The cohort comprised 14,810 women, with 780 receiving cabergoline within one month of childbirth and 13,898 serving as the control group. The epidemiological characteristics of these groups are detailed in **Supplementary Table S17**.

**Figure 7:**
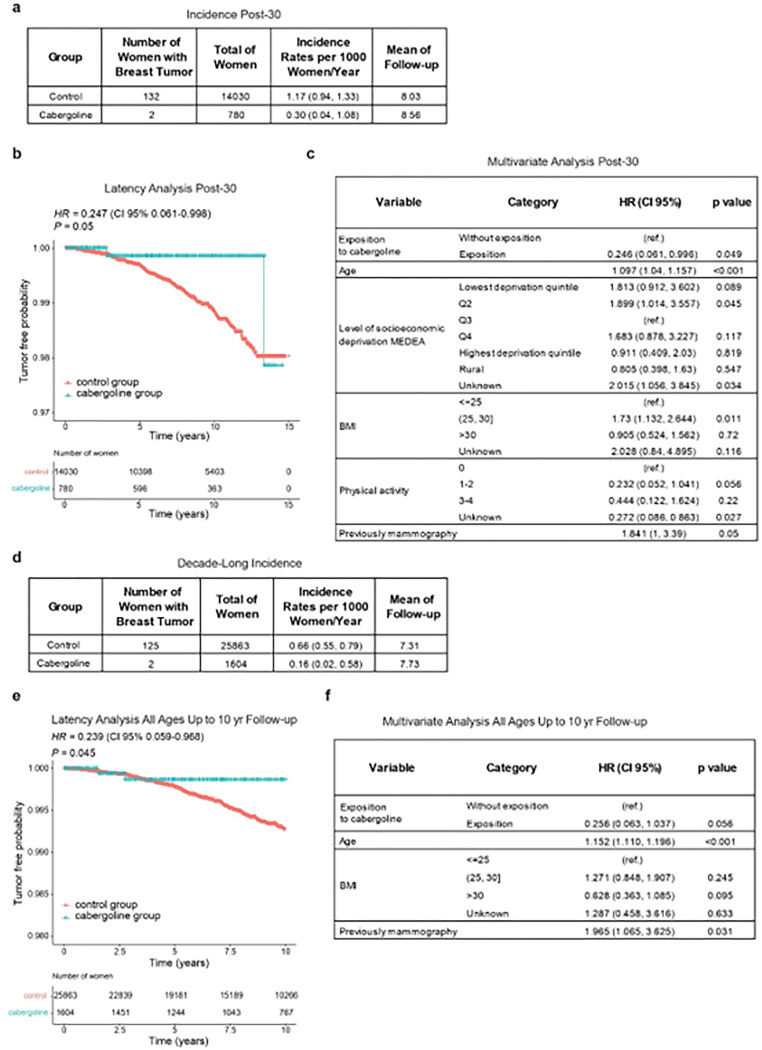
Evaluation of post-pregnancy cabergoline administration on breast cancer risk in women. **a**) Cumulative incidence in older first-time mothers: analyzes the cumulative incidence of breast cancer in women who had their first child at age 30 or older, comparing those who received cabergoline post-pregnancy with controls. **b**) Kaplan-Meier survival analysis and HR in older first-time mothers: displays the Kaplan-Meier survival curve and estimates Hazard Ratio (HR) using bivariate Cox regression for the same cohort. **c**) Multivariate Cox regression in older first-time mothers: assesses the influence of cabergoline intake and other variables on breast cancer risk using a multivariate Cox regression model. **d**) Cumulative incidence in a decade post-first pregnancy: shows the cumulative incidence of breast cancer within ten years following the first pregnancy in a broader cohort. **e**) Kaplan-Meier curve and HR post-first pregnancy: Kaplan-Meier survival curve with HR estimated by bivariate Cox regression for the broader cohort. **f**) Multivariate Cox regression analysis in a decade post-first pregnancy: examines breast cancer risk factors post-first pregnancy, including cabergoline intake, through multivariate Cox regression analysis.

In the control group, 132 breast cancer cases were documented, contrasting with only 2 cases in the cabergoline-treated group. The annual cumulative incidence rates per 1,000 population were 1.17 for the control group and 0.3 for the treated group, with average follow-up durations of 8.03 and 8.56 years, respectively (Fig. 7a). Bivariate Cox regression yielded a Hazard Ratio (HR) of 0.247 (95% CI: 0.061 – 0.998, P = 0.050) for the cabergoline-treated group (Fig. 7b), with additional bivariate analysis details in **Supplementary Table S18**. Further, a multivariate Cox regression, adjusting for significant epidemiological breast cancer risk factors, substantiated the diminished risk in the cabergoline-treated group (HR = 0.239; 95% CI: 0.059 – 0.968) (Fig. 7c).

Expanding the scope, we examined breast cancer incidence over a decade following the first pregnancy, irrespective of maternal age. This included 27,467 women, with 1,604 in the cabergoline-treated group and 25,863 controls. The control group reported 125 breast cancer cases, compared to just 2 in the treated group. Detailed epidemiological profiles are available in **Supplementary Table S19**. The annual cumulative incidence rates per 1,000 population were 0.66 for the control group and 0.17 for the treated group (Fig. 7d). Bivariate Cox regression analysis indicated an HR of 0.239 (95% CI: 0.059 – 0.968, P = 0.045) for the cabergoline group (Fig. 7e), with further information in **Supplementary Table S20**. The multivariate Cox regression analysis reaffirmed a consistently lower breast cancer risk in the cabergoline-treated group (adjusted P = 0.056) (Fig. 7f).

In summary, these results collectively suggest that administering cabergoline postpartum may significantly reduce the risk of breast cancer following pregnancy, highlighting its potential as a protective intervention in this specific context.

## DISCUSSION

The escalating incidence of breast cancer, particularly in the context of delayed childbearing and the specific challenges of post-pregnancy breast cancer, underscores the pressing need for novel chemopreventive strategies (1–4). Our study breaks new ground by proposing cabergoline, a dopaminergic agonist, as a potential pharmacological agent for breast cancer prevention, notably in the post-pregnancy period.

This research demonstrates the efficacy of cabergoline in enhancing postlactational involution, subsequently reducing breast cancer risk in *Brca1/P53*-deficient mice. The significance of this finding is amplified by the increasing trend of delayed childbirth (7) and its correlation with heightened breast cancer risk, particularly among women aged over 30–35 years (4). The search for new pharmacological alternatives is further emphasized by the limitations and adverse effects associated with current chemopreventive methods such as selective estrogen receptor modulators (SERMs), aromatase inhibitors (AIs), and surgical options (9–11). Cabergoline emerges as a promising candidate, offering potential for wider application with fewer side effects (43, 44).

Our findings are particularly relevant considering that approximately 80% of mammary epithelial cells undergo apoptosis during postlactational involution, including those with genetic mutations (102, 103). The age-related accumulation of premalignant lesions, if not cleared, may lead to cancer initiation (104, 105). Cabergoline-induced intensification of postlactational involution aligns with a decrease in long-term tumor susceptibility, a crucial consideration given the pro-inflammatory environment of postlactational involution, which could otherwise promote tumorigenesis (106).

Importantly, our study demonstrates that cabergoline not only induces a hypoproliferative state in mammary epithelial tissue but also mitigates the glandular component, which correlates positively with mammographic density, a well-documented risk factor for breast cancer (59, 61, 94). RNA sequencing and enrichment analyses have further substantiated cabergoline’s role in activating antiproliferative pathways, underscoring its preventive potential.

Moreover, we highlight the impact of cabergoline on reducing the fraction of *Brca1/P53*-deficient recombinant cells in the mammary gland. Drawing on the concept of “field cancerization” (94), our findings indicate that cabergoline-treated mice exhibit fewer pre-malignant cells. This reduction, paired with the diminished glandular component, suggests that cabergoline narrows the ‘field cancerization’ zone, amplifying the protective shield against breast cancer established post-pregnancy.

Extending to human applications, our epidemiological study suggests cabergoline’s potential in reducing post-pregnancy breast cancer risk. This is especially pivotal given the current absence of targeted chemopreventive strategies for this subtype of cancer (8). While our data do not distinguish between ER-negative and ER-positive tumors, they hint at cabergoline’s potential efficacy against ER-negative breast cancer, typically more common in younger women (107).

However, our study has limitations. The epidemiological component confirmed breast cancer prevention only in post-pregnancy cases. Further analysis, possibly including cohorts treated with cabergoline prior to 2005, is needed to ascertain cabergoline’s long-term effects on postmenopausal breast cancer. Additional studies are required to confirm the long-term implications of cabergoline on breast cancer risk.

Cabergoline, typically used in a single dose for lactation cessation, is known for its tolerability (43, 44). Its minimal side effects and convenient dosing present an advantageous alternative to current chemopreventive methods that often require daily intake over extended periods (9, 14, 108).

In conclusion, our study introduces cabergoline as a novel and potentially effective strategy for breast cancer prevention, particularly in the post-pregnancy context. Its application post-breastfeeding in women at high risk of breast cancer could enhance the natural protective effect of pregnancy. Cabergoline’s potential to prevent both hormone receptor-negative and post-pregnancy breast cancer could represent a significant advancement in breast cancer prevention strategies. Further research is warranted to explore its long-term effects and suitability for a broader population.

## METHODS

### Mice models and experimental procedures

#### Model development:

Utilizing genetically modified mice, our study employed the K-14 *Cre; Brca1*^*f/f*^*/P53*^*f/f*^ *(Brca1/P53)* double knockout model, expressing Cre recombinase under the cytokeratin 14 promoter for targeted Brca1 and P53 deletion in specific cells (45). Additionally, we integrated the *RFP; K-14 Cre; Brca1*^*f/f*^*/P53*^*f/f*^ model, established by crossing *Brca1/P53* mice with *C57BL/6-Gt(ROSA)26tm1Hjf* mice (97). All models were housed at the University of Salamanca’s Animal Research Facility, adhering to ethical and pathogen-free standards. In the different studies, female subjects started mating at the age of six weeks.

#### Study design:

The investigation was segmented into three core studies:

#### Early postlactational analysis:

Focusing on early post-weaning phases (1, 3, and 7 days), we evaluated mammary gland involution in *Brca1/P53*-deficient and wild-type mice, both treated and untreated with cabergoline. Each subgroup consisted of five mice.

#### Longitudinal tumor susceptibility:

This segment tracked breast tumor development in female *Brca1/P53*-deficient mice, categorized into various groups based on parity and cabergoline treatment, totaling 169 subjects, segmented into 35 nulliparous, 32 multiparous, 37 cabergoline-treated multiparous, 37 cabergoline-treated nulliparous, and 28 bi-weekly treated nulliparous mice. We monitored them weekly for tumor emergence, applying humane euthanasia protocols for those meeting predefined criteria.

#### Cross-sectional mammary assessment:

We analyzed mammary gland status in both *Brca1/P53*-deficient and wild-type mice, post-lactational involution (30 and 60 days), and in age-matched nulliparous counterparts, with and without cabergoline treatment.

#### Genotyping procedures:

Utilizing PCR, we identified the RFP+ transgene and *Brca/P53* deletion in tail DNA samples. The PCR protocol involved specific reagents, cycling conditions, and oligonucleotide sequences previously reported (45, 97), with final products analyzed on 1% agarose gels.

#### Cabergoline administration:

Mimicking human dosage for lactation inhibition (109, 110), cabergoline was prepared in a saline-methylcellulose solution and administered intraperitoneally at 0.25 mg/kg. Dosage adjustments were based on individual weights, with administration under isofluorane anesthesia.

#### Tumor susceptibility monitoring:

Weekly palpation sessions were conducted to detect primary mammary tumors, recording both tumor latency and incidence. Euthanasia was carried out upon signs of sickness, rapid tumor growth, or wound development, ensuring adherence to ethical guidelines.

### Histological and immunohistochemical analyses

#### Histological analysis of mammary glands:

Mammary glands were fixed in 4% paraformaldehyde (PFA) and processed for paraffin embedding (Shandon Excelsior, Thermo). Tissue sections were stained with hematoxylin and eosin for morphological examination. Quantitative analysis of adipocyte and ductal areas was conducted on five randomly selected images at 10x magnification per sample using a Leica ICC50 HD camera and Leica Application Suite V3.7 software. ImageJ was employed to determine the relative ductal epithelial area as a percentage of the total field area.

#### Ki-67 and cleaved-caspase 3 immunohistochemistry:

For cell proliferation and apoptosis assessment, 3 μm sections of mammary glands and tumors were stained with Ki-67 (MAD020310Q, 1:50 dilution, Master Diagnostica) and cleaved caspase-3 (Asp175) antibodies (#9661, Cell Signaling). Immunostaining was performed using the Discovery ULTRA system (Roche) with OmniMap anti-Rb HRP secondary antibody (#05269679001, Roche). Quantification of positive cells in five selected areas at 20x magnification per slide was done using Leica Application Suite V3.7 software.

#### Quantification of caspase-3 and Ki-67 positive cells:

Five random images of the distal area of each mammary gland were captured at 20X magnification (Leica ICC50 HD camera, Leica DM750 microscope). The Leica Application Suite V3.7 was used to identify and count positive and negative cells. The proportion of positive cells was calculated as the number of positive cells divided by the total number of cells, multiplied by 100.

### Immunohistochemical detection of RFP

For the detection of red fluorescent protein (RFP), tissue sections (3 μm) were prepared on Superfrost^®^ Plus slides and air-dried overnight. Immunohistochemistry was conducted using the automated Discovery ULTRA system (Ventana-Roche). Antigen retrieval was achieved using CC1 buffer (Ventana, Roche), and endogenous peroxidase activity was blocked using 3% hydrogen peroxide.

Sections were incubated with a rabbit polyclonal anti-TagRFP primary antibody (1:2500, Invitrogen, Cat# R10367). This was followed by incubation with an OmniMap anti-rabbit HRP-conjugated secondary system (Ventana, Roche, Cat# 760-4311). Visualization was achieved using the ChromoMap DAB kit (Ventana, Roche).

### Organoid generation from mouse mammary glands

Organoids enriched in epithelial cells were generated following the protocol by Smalley (111). Mammary glands were mechanically fragmented and treated with DMEM/F-12, fetal bovine serum, and antibiotics, supplemented with collagenase (#C2674, Sigma-Aldrich) and trypsin (#15090046, Thermo Fisher Scientific). Post-incubation, the epithelial cell-enriched pellet was washed with PBS containing fetal bovine serum and DNase (#DN25, Sigma-Aldrich), and subsequently aliquoted and stored at −80°C for RNA and DNA extractions.

### Quantitative PCR for *P53* allele and normalization

For quantifying the recombinant P53 allele in DNA from organoids, we performed qPCR. The reaction included DNA (20 ng/ml), P53del-specific oligonucleotides (forward: 5’-GAGACGGAGAAAGGGCGACT-3’; reverse: 5’-CTAGAACTAGTGGATCCCCCG-3’) at 25 μM, Perfecta Sybr Green SuperMix ROX (QuantaBio, #95055-500), and water. GAPDH (forward: 5’-CTGCACCACCAACTGCTTAG-3’; reverse: 5’-GTCTTCTGGGTGGCAGTGAT-3’) was the reference gene. The PCR protocol started with a 10-minute denaturation at 95°C, followed by 40 cycles. Fluorescence detection used the Mastercycler ep Realplex 2 (Eppendorf). Gene expression was analyzed in triplicate and quantified using the ΔΔCt method. Normalization involved adjusting the relative quantity (RQ) of each sample for the ductal area by multiplying the RQ by the mean ductal area percentage (from five randomly selected fields) and then dividing by 100.

### Protein analysis in organoid samples

Protein extraction from organoids used RIPA buffer, with protease and phosphatase inhibitor cocktails (#P8340, Sigma Aldrich), was carried out as previously (28). Post-extraction, protein concentration was determined using the Bradford Protein Assay (#5000006, Bio-Rad). Electrophoretic separation utilized SDS-PAGE on 10% or 12% gels, followed by transfer to PVDF membranes (Immobilon-P, Millipore). The membranes were probed with primary antibodies: pSTAT5 (1:1000, #9351, Cell Signaling), STAT5 (1:1000, #13179-1-AP, Proteintech), pSTAT3 (1:1000, #9145, Cell Signaling), STAT3 (1:1000, #4904, Cell Signaling), pERK1/2 (1:1000, #9101, Cell Signaling), pAKT S473 (1:1000, #4058, Cell Signaling), GAPDH (1:1000, #60004-1-Ig, Proteintech), and actin (1:1000, #A5441, Sigma‒Aldrich). Detection was through enhanced chemiluminescence (ECL, #170-5061, Bio-Rad) and imaging on an ImageQuant LAS 500 (GE Healthcare Life Sciences).

### RNA extraction and library preparation for sequencing

RNA was extracted from organoids using the Qiagen miRNeasy Mini Kit. Quality and integrity assessments involved agarose gel electrophoresis, Nanodrop, and an Agilent 5400 bioanalyzer. Library construction for RNA sequencing was conducted by Novogene, including rRNA removal, RNA fragmentation, cDNA synthesis, and adapter ligation, with sequencing on the Illumina NovaSeq 6000 PE150. Data is available under GSE250534 in the Gene Ontology database.

### Statistical analysis of RNA-seq data

Analysis of RNA-Seq data by the Bioinformatics Department at Salamanca Cancer Research Center included quality control with RaNA-seq (112), gene expression quantification using Salmon (v0.9.1) (113), differential expression analysis with edgeR (v3.24) (114), and over-representation analysis using clusterProfiler (v4.6.0) (115, 116). Gene set similarities were assessed using the Jaccard similarity index, and K-means clustering was applied for data categorization, using the elbow method for optimization.

### Mammary gland processing and flow cytometry analysis

#### Dissociation of mammary glands:

Employing *Brca1/P53*-deficient mice with Cre-recombinase (45) crossed with RFP-bearing mice (97), we prepared mammary glands for single-cell analysis. Glands were dissected, avoiding lymph nodes, and incubated with collagenase (#C2674, Sigma Aldrich, 6000 U/ml) and hyaluronidase (#H3506, Sigma Aldrich, 2000 U/ml) in DMEM/F-12 medium (#11554546, Thermo Fisher Scientific). The glands were mechanically disrupted post-incubation and the cell pellet obtained through centrifugation. Red blood cells were lysed using ACK buffer (#A10492-01, Thermo Fisher Scientific), followed by trypsinization to dissociate cells. DNase treatment (#DN25, Sigma-Aldrich) was applied, and cell suspensions were filtered (40 μm filter, #352340, Corning) to remove aggregates.

#### Flow cytometry of epithelial subpopulations:

Cell suspensions were blocked with anti-CD16/CD32 antibody (#MO16PU (V500), ImmunoStep) and stained with a panel of antibodies: CD45 (FITC, 1:100, #553080, BD Biosciences), CD140 (FITC, 1:100, #11-1401-82, Invitrogen), CD31 (FITC, 1:100, #553372, BD Biosciences), Ter119 (FITC, 1:100, #557915, BD Biosciences), CD49f (APC, 1:100, #17-0495-80, eBioscience), CD61 (BV421, 1:100, #566227, BD Biosciences), Sca1 (PerCP-Cy5, 1:100, #45-5981-80, Invitrogen), EpCAM (PE-Cy7, 1:200, #M326PC7, ImmunoStep), and eFluor^™^ 506 (1:100, #65-0866-14, Invitrogen). Cells negative for lineage markers (Lin-) – including leukocytes, erythroid cells, endothelial cells, and fibroblasts – were identified and excluded based on the absence of CD45, TER119, CD34, and CD140 markers. After staining, cells were treated with Fixable Viability Dye eFluor^™^ 506 and analyzed on the LSR Fortessa X-20 Cell Analyzer (BD Biosciences) using FlowJo V10 software (Treestar, California).

#### Normalization for ductal area:

Each epithelial cell population’s percentage was normalized against the mean ductal area specific to each mouse, calculated from five randomly selected fields. The normalized value for each population was computed by multiplying its percentage by the mean ductal area percentage and dividing by 100.

### Epidemiological study design and data analysis in SIDIAP database

#### Study design and cohort selection:

We conducted a retrospective cohort study using the SIDIAP (Information System for the Improvement of Research in Primary Care) (Catalunya, Spain) database, encompassing over 80% of Catalonia’s population from 2005 onwards, to evaluate the impact of cabergoline on breast cancer prevention (100, 101). The study, authorized by the SIDIAP Scientific and Ethics Committees, included women who had pregnancies lasting between 35 and 50 weeks. This range was chosen based on existing evidence of pregnancy’s protective effect against breast cancer starting at 35 weeks (117).

#### Inclusion criteria and data collection:

The cohort comprised women with detailed pregnancy records, including dates of the last menstrual period and pregnancy outcomes. From the 123,422 eligible pregnancies, 5,472 women who took cabergoline during or immediately after pregnancy formed the treatment group, while 102,959 women served as controls. Exclusions were made for cabergoline use unrelated to pregnancy and previous breast cancer diagnoses (**Extended Data Fig. 7**).

#### Variables and sociodemographic factors:

The study considered various factors, including age, socioeconomic status (MEDEA index), term and premature births, and pregnancy duration. Clinical variables encompassed health conditions, family cancer history, and lifestyle factors such as tobacco use and physical activity **(Supplementary Tables S17, S19**).

#### Epidemiological and statistical analysis:

Analysis was segmented into two groups based on age at first pregnancy and follow-up duration post-pregnancy. Descriptive statistics were generated, with Fisher’s exact test and Student’s t-test employed for comparing categorical and quantitative variables, respectively. Breast cancer incidence rates per 1000 inhabitants were calculated annually. Kaplan-Meier curves, supplemented by log-rank tests, assessed temporal patterns like cancer onset latency. Cox regression models, both bivariate and multivariate (using the Wald method), were used to estimate hazard ratios (HR) and 95% confidence intervals (CI), focusing on variables significantly associated with breast cancer. All tests were two-tailed with a 5% significance level, analyzed using R 3.2.1. (R Foundation for Statistical Computing, Vienna, Austria), with SIDIAP’s data analysts leading the analysis.

### Supplementary statistical methods

No statistical method was used to predetermine the sample size. The experiments were not randomized. Investigators were blinded during the study analyses. For the various variables studied, their distribution was examined, and conformity to a normal distribution was determined using the Shapiro-Wilk test. Depending on data distribution, Pearson or Spearman correlation coefficients were calculated. For comparisons between two groups, Student’s t-test or Mann–Whitney U test were applied, while ANOVA or Kruskal–Wallis tests were used for analyses involving more than two groups. Kaplan–Meier estimators and log-rank tests compared temporal variables. Fisher’s exact test and chi-square tests were utilized for contingency analysis. Graphpad Prism 8 and JMP12 were used for statistical analyses.

## Data Availability

**RNA-Seq Data Accessibility**: The RNA sequencing data generated during the current study are available in the Gene Expression Omnibus (GEO) database, under accession number GSE250534. This dataset is open for access by the broader scientific community. **RNA-Seq Data Accessibility**: Extensive tables detailing the descriptive analyses conducted in our human study are available on the Figshare Repository. These tables provide comprehensive insights into various aspects of our research, supplementing the findings presented in this manuscript. The data can be accessed at https://doi.org/10.6084/m9.figshare.24967416 **Restrictions on Raw Human Data**: The raw human data utilized in this study were obtained from the Catalonian Health Institute’s Information System for the Development of Research in Primary Care (SIDIAP). Access to the raw data is limited under the data-sharing agreement with SIDIAP, which allows only the researchers involved in this study to access the data, in accordance with European and national data protection regulations. **Access to SIDIAP Data**: Researchers affiliated with public institutions who wish to access the SIDIAP data for legitimate scientific purposes may request such access. This request is subject to stringent criteria, including ethical and scientific approval by the Ethics and Clinical Research Committee of the Primary Care Research Institute Jordi Gol/SIDIAP Jordi Gol. Interested researchers can find more information about the data request procedures and contact details at the SIDIAP website or by reaching out to SIDIAP at sidiap@idiapjgol.org.

## References

[1] Sung H, Ferlay J, Siegel RL, Laversanne M, Soerjomataram I, Jemal A, Global Cancer Statistics 2020: GLOBOCAN Estimates of Incidence and Mortality Worldwide for 36 Cancers in 185 Countries. CA Cancer J Clin. 2021;71(3):209–49. 33538338 10.3322/caac.21660

[2] Global Burden of Disease Cancer C, Fitzmaurice C, Abate D, Abbasi N, Abbastabar H, Abd-Allah F, Global, Regional, and National Cancer Incidence, Mortality, Years of Life Lost, Years Lived With Disability, and Disability-Adjusted Life-Years for 29 Cancer Groups, 1990 to 2017: A Systematic Analysis for the Global Burden of Disease Study. JAMA Oncol. 2019;5(12):1749–68. 31560378 10.1001/jamaoncol.2019.2996 PMC6777271

[3] Ginsburg O, Bray F, Coleman MP, Vanderpuye V, Eniu A, Kotha SR, The global burden of women’s cancers: a grand challenge in global health. Lancet. 2017;389(10071):847–60. 27814965 10.1016/S0140-6736(16)31392-7 PMC6191029

[4] Nichols HB, Schoemaker MJ, Cai J, Xu J, Wright LB, Brook MN, Breast Cancer Risk After Recent Childbirth: A Pooled Analysis of 15 Prospective Studies. Ann Intern Med. 2019;170(1):22–30. 30534999 10.7326/M18-1323 PMC6760671

[5] Giaquinto AN, Sung H, Miller KD, Kramer JL, Newman LA, Minihan A, Breast cancer statistics, 2022. CA: a cancer journal for clinicians. 2022;72(6):524–41. 36190501 10.3322/caac.21754

[6] Lambe M, Hsieh C-c, Trichopoulos D, Ekbom A, Pavia M, Adami H-O. Transient increase in the risk of breast cancer after giving birth. New England Journal of Medicine. 1994;331(1):5–9. 8202106 10.1056/NEJM199407073310102

[7] Martin JA, Hamilton BE, Osterman MJK, Driscoll AK. Births: Final Data for 2018. Natl Vital Stat Rep. 2019;68(13):1–47. 32501202

[8] Zhang Z, Bassale S, Jindal S, Fraser A, Guinto E, Anderson W, Young-Onset Breast Cancer Outcomes by Time Since Recent Childbirth in Utah. JAMA Netw Open. 2022;5(10):e2236763. 36239933 10.1001/jamanetworkopen.2022.36763 PMC9568799

[9] Nelson HD, Fu R, Zakher B, Pappas M, McDonagh M. Medication Use for the Risk Reduction of Primary Breast Cancer in Women: Updated Evidence Report and Systematic Review for the US Preventive Services Task Force. JAMA. 2019;322(9):868–86. 31479143 10.1001/jama.2019.5780

[10] Hartmann LC, Schaid DJ, Woods JE, Crotty TP, Myers JL, Arnold PG, Efficacy of bilateral prophylactic mastectomy in women with a family history of breast cancer. N Engl J Med. 1999;340(2):77–84. 9887158 10.1056/NEJM199901143400201

[11] Rebbeck TR, Friebel T, Lynch HT, Neuhausen SL, van ‘t Veer L, Garber JE, Bilateral prophylactic mastectomy reduces breast cancer risk in BRCA1 and BRCA2 mutation carriers: the PROSE Study Group. J Clin Oncol. 2004;22(6):1055–62. 14981104 10.1200/JCO.2004.04.188

[12] Carbine NE, Lostumbo L, Wallace J, Ko H. Risk-reducing mastectomy for the prevention of primary breast cancer. Cochrane Database Syst Rev. 2018;4(4):CD002748. 29620792 10.1002/14651858.CD002748.pub4 PMC6494635

[13] Owens DK, Davidson KW, Krist AH, Barry MJ, Cabana M, Caughey AB, Medication use to reduce risk of breast cancer: US Preventive Services Task Force recommendation statement. Jama. 2019;322(9):857–67. 31479144 10.1001/jama.2019.11885

[14] Roetzheim RG, Lee JH, Fulp W, Matos Gomez E, Clayton E, Tollin S, Acceptance and adherence to chemoprevention among women at increased risk of breast cancer. Breast. 2015;24(1):51–6. 25491191 10.1016/j.breast.2014.11.006 PMC4503358

[15] Uray IP, Brown PH. Chemoprevention of hormone receptor-negative breast cancer: new approaches needed. Clinical Cancer Prevention. 2011:147–62. 10.1007/978-3-642-10858-7_13 PMC3415693 21253797

[16] MacMahon B, Cole P, Lin TM, Lowe CR, Mirra AP, Ravnihar B, Age at first birth and breast cancer risk. Bull World Health Organ. 1970;43(2):209–21. 5312521 PMC2427645

[17] Ma H, Bernstein L, Pike MC, Ursin G. Reproductive factors and breast cancer risk according to joint estrogen and progesterone receptor status: a meta-analysis of epidemiological studies. Breast Cancer Research. 2006;8(4):1–11. 10.1186/bcr1525 PMC1779465 16859501

[18] Andrieu N, Goldgar DE, Easton DF, Rookus M, Brohet R, Antoniou AC, Pregnancies, breast-feeding, and breast cancer risk in the International BRCA1/2 Carrier Cohort Study (IBCCS). Journal of the National Cancer Institute. 2006;98(8):535–44. 16622123 10.1093/jnci/djj132 PMC2094011

[19] Evans D, Harkness E, Howel S, Woodward E, Howell A, Lalloo F. Young age at first pregnancy does protect against early onset breast cancer in BRCA1 and BRCA2 mutation carriers. Breast cancer research and treatment. 2018;167:779–85. 29116468 10.1007/s10549-017-4557-1 PMC5807493

[20] Faupel-Badger JM, Arcaro KF, Balkam JJ, Eliassen AH, Hassiotou F, Lebrilla CB, Postpartum remodeling, lactation, and breast cancer risk: summary of a National Cancer Institute–sponsored workshop. Journal of the National Cancer Institute. 2013;105(3):166–74. 23264680 10.1093/jnci/djs505 PMC3611853

[21] Marchant J. Influence of pregnancy and lactation on the incidence of mammary carcinoma induced with methylcholanthrene in female mice of the “IF” strain. J Pathol Bacteriol. 1955;70(2):415–8. 13295916 10.1002/path.1700700218

[22] Sinha DK, Pazik JE, Dao TL. Prevention of mammary carcinogenesis in rats by pregnancy: effect of full-term and interrupted pregnancy. Br J Cancer. 1988;57(4):390–4. 3134040 10.1038/bjc.1988.88 PMC2246553

[23] Rajkumar L, Kittrell FS, Guzman RC, Brown PH, Nandi S, Medina D. Hormone-induced protection of mammary tumorigenesis in genetically engineered mouse models. Breast Cancer Res. 2007;9(1):R12. 17257424 10.1186/bcr1645 PMC1851398

[24] Meier-Abt F, Bentires-Alj M, Rochlitz C. Breast cancer prevention: lessons to be learned from mechanisms of early pregnancy–mediated breast cancer protection. Cancer research. 2015;75(5):803–7. 25660950 10.1158/0008-5472.CAN-14-2717

[25] Slepicka PF, Cyrill SL, Dos Santos CO. Pregnancy and Breast Cancer: Pathways to Understand Risk and Prevention. Trends Mol Med. 2019;25(10):866–81. 31383623 10.1016/j.molmed.2019.06.003

[26] Feigman MJ, Moss MA, Chen C, Cyrill SL, Ciccone MF, Trousdell MC, Pregnancy reprograms the epigenome of mammary epithelial cells and blocks the development of premalignant lesions. Nature communications. 2020;11(1):2649. 10.1038/s41467-020-16479-z PMC7253414 32461571

[27] Radisky DC, Hartmann LC. Mammary involution and breast cancer risk: transgenic models and clinical studies. J Mammary Gland Biol Neoplasia. 2009;14(2):181–91. 19404726 10.1007/s10911-009-9123-y PMC2693781

[28] Castillo-Lluva S, Hontecillas-Prieto L, Blanco-Gómez A, del Mar Sáez-Freire M, García-Cenador B, García-Criado J, A new role of SNAI2 in postlactational involution of the mammary gland links it to luminal breast cancer development. Oncogene. 2015;34(36):4777–90. 26096931 10.1038/onc.2015.224 PMC4560637

[29] JaÈger R, Herzer U, Schenkel J, Weiher H. Overexpression of Bcl-2 inhibits alveolar cell apoptosis during involution and accelerates c-myc-induced tumorigenesis of the mammary gland in transgenic mice. Oncogene. 1997;15(15):1787–95. 9362445 10.1038/sj.onc.1201353

[30] Schorr K, Li M, Bar-Peled U, Lewis A, Heredia A, Lewis B, Gain of Bcl-2 is more potent than bax loss in regulating mammary epithelial cell survival in vivo. Cancer research. 1999;59(11):2541–5. 10363969

[31] Furth PA, Bar-Peled U, Li M, Lewis A, Laucirica R, Jäger R, Loss of anti-mitotic effects of Bcl-2 with retention of anti-apoptotic activity during tumor progression in a mouse model. Oncogene. 1999;18(47):6589–96. 10597263 10.1038/sj.onc.1203073

[32] Hutchinson J, Jin J, Cardiff RD, Woodgett JR, Muller WJ. Activation of Akt (protein kinase B) in mammary epithelium provides a critical cell survival signal required for tumor progression. Molecular and cellular biology. 2001;21(6):2203–12. 11238953 10.1128/MCB.21.6.2203-2212.2001 PMC86854

[33] Ackler S, Ahmad S, Tobias C, Johnson MD, Glazer RI. Delayed mammary gland involution in MMTV-AKT1 transgenic mice. Oncogene. 2002;21(2):198–206. 11803463 10.1038/sj.onc.1205052

[34] Hutchinson JN, Jin J, Cardiff RD, Woodgett JR, Muller WJ. Activation of Akt-1 (PKB-α) can accelerate ErbB-2-mediated mammary tumorigenesis but suppresses tumor invasion. Cancer research. 2004;64(9):3171–8. 15126356 10.1158/0008-5472.can-03-3465

[35] Shibata M-A, Liu M-L, Knudson MC, Shibata E, Yoshidome K, Bandey T, Haploid loss of bax leads to accelerated mammary tumor development in C3 (1)/SV40-TAg transgenic mice: reduction in protective apoptotic response at the preneoplastic stage. The EMBO Journal. 1999;18(10):2692–701. 10329616 10.1093/emboj/18.10.2692 PMC1171351

[36] Maroulakou IG, Oemler W, Naber SP, Tsichlis PN. Akt1 ablation inhibits, whereas Akt2 ablation accelerates, the development of mammary adenocarcinomas in mouse mammary tumor virus (MMTV)-ErbB2/neu and MMTV-polyoma middle T transgenic mice. Cancer research. 2007;67(1):167–77. 17210696 10.1158/0008-5472.CAN-06-3782

[37] Maroulakou IG, Oemler W, Naber SP, Klebba I, Kuperwasser C, Tsichlis PN. Distinct roles of the three Akt isoforms in lactogenic differentiation and involution. Journal of cellular physiology. 2008;217(2):468–77. 18561256 10.1002/jcp.21518 PMC2871282

[38] Humphreys RC, Hennighausen L. Signal transducer and activator of transcription 5a influences mammary epithelial cell survival and tumorigenesis. 1999. 10547072

[39] Ren S, Cai HR, Li M, Furth PA. Loss of Stat5a delays mammary cancer progression in a mouse model. Oncogene. 2002;21(27):4335–9. 12082622 10.1038/sj.onc.1205484

[40] Zhao X, Ponchon B, Lanctôt S, Lacasse P. Invited review: Accelerating mammary gland involution after drying-off in dairy cattle. Journal of dairy science. 2019;102(8):6701–17. 31202662 10.3168/jds.2019-16377

[41] Boutinaud M, Isaka N, Lollivier V, Dessauge F, Gandemer E, Lamberton P, Cabergoline inhibits prolactin secretion and accelerates involution in dairy cows after dry-off. J Dairy Sci. 2016;99(7):5707–18. 27179868 10.3168/jds.2015-10782

[42] Boutinaud M, Isaka N, Gandemer E, Lamberton P, Wiart S, Taranilla AIP, Inhibiting prolactin by cabergoline accelerates mammary gland remodeling during the early dry period in dairy cows. J Dairy Sci. 2017;100(12):9787–98. 28964519 10.3168/jds.2017-12783

[43] Yang Y, Boucoiran I, Tulloch KJ, Poliquin V. Is Cabergoline Safe and Effective for Postpartum Lactation Inhibition? A Systematic Review. Int J Womens Health. 2020;12:159–70. 32210637 10.2147/IJWH.S232693 PMC7069562

[44] Boucoiran I, Roy M, Poliquin V, Elwood C, Sheehan NL, Thibaudeau R, Evaluation of cabergoline for lactation inhibition in women living with HIV. Int J STD AIDS. 2021;32(7):654–61. 33612017 10.1177/0956462420984694

[45] Liu X, Holstege H, van der Gulden H, Treur-Mulder M, Zevenhoven J, Velds A, Somatic loss of BRCA1 and p53 in mice induces mammary tumors with features of human BRCA1-mutated basal-like breast cancer. Proc Natl Acad Sci U S A. 2007;104(29):12111–6. 17626182 10.1073/pnas.0702969104 PMC1924557

[46] Hughes K, Watson CJ. The Multifaceted Role of STAT3 in Mammary Gland Involution and Breast Cancer. Int J Mol Sci. 2018;19(6). 10.3390/ijms19061695 PMC6032292 29875329

[47] Subramanian A, Tamayo P, Mootha VK, Mukherjee S, Ebert BL, Gillette MA, Gene set enrichment analysis: a knowledge-based approach for interpreting genome-wide expression profiles. Proc Natl Acad Sci U S A. 2005;102(43):15545–50. 16199517 10.1073/pnas.0506580102 PMC1239896

[48] Goeman JJ, Buhlmann P. Analyzing gene expression data in terms of gene sets: methodological issues. Bioinformatics. 2007;23(8):980–7. 17303618 10.1093/bioinformatics/btm051

[49] Gundogdu P, Loucera C, Alamo-Alvarez I, Dopazo J, Nepomuceno I. Integrating pathway knowledge with deep neural networks to reduce the dimensionality in single-cell RNA-seq data. BioData Min. 2022;15(1):1. 34980200 10.1186/s13040-021-00285-4 PMC8722116

[50] Cai J, Zhan J, Arking DE, Bader JS. Priors, population sizes, and power in genome-wide hypothesis tests. BMC Bioinformatics. 2023;24(1):170. 37101120 10.1186/s12859-023-05261-9 PMC10134629

[51] Ghanbari M, Lasserre J, Vingron M. Reconstruction of gene networks using prior knowledge. BMC Syst Biol. 2015;9:84. 26589494 10.1186/s12918-015-0233-4 PMC4654848

[52] Kondratskyi A, Kondratska K, Skryma R, Prevarskaya N. Ion channels in the regulation of apoptosis. Biochim Biophys Acta. 2015;1848(10 Pt B):2532–46. 25450339 10.1016/j.bbamem.2014.10.030

[53] Davis FM. The ins and outs of calcium signalling in lactation and involution: Implications for breast cancer treatment. Pharmacol Res. 2017;116:100–4. 27965034 10.1016/j.phrs.2016.12.007

[54] Reinhardt TA, Lippolis JD. Mammary gland involution is associated with rapid down regulation of major mammary Ca2+-ATPases. Biochem Biophys Res Commun. 2009;378(1):99–102. 19000904 10.1016/j.bbrc.2008.11.004

[55] VanHouten J, Sullivan C, Bazinet C, Ryoo T, Camp R, Rimm DL, PMCA2 regulates apoptosis during mammary gland involution and predicts outcome in breast cancer. Proc Natl Acad Sci U S A. 2010;107(25):11405–10. 20534448 10.1073/pnas.0911186107 PMC2895115

[56] Arnandis T, Ferrer-Vicens I, Garcia-Trevijano ER, Miralles VJ, Garcia C, Torres L, Calpains mediate epithelial-cell death during mammary gland involution: mitochondria and lysosomal destabilization. Cell Death Differ. 2012;19(9):1536–48. 22555453 10.1038/cdd.2012.46 PMC3422479

[57] Jeong J, Lee J, Talaia G, Kim W, Song J, Hong J, Intracellular Calcium links Milk Stasis to Lysosome Dependent Cell Death by Activating a TGFbeta3/TFEB/STAT3 Pathway Early during Mammary Gland Involution. Res Sq. 2023.

[58] Zhai M, Yang D, Yi W, Sun W. Involvement of calcium channels in the regulation of adipogenesis. Adipocyte. 2020;9(1):132–41. 32175809 10.1080/21623945.2020.1738792 PMC7153653

[59] Boyd NF, Guo H, Martin LJ, Sun L, Stone J, Fishell E, Mammographic density and the risk and detection of breast cancer. New England journal of medicine. 2007;356(3):227–36. 17229950 10.1056/NEJMoa062790

[60] Weaver O, Leung JWT. Biomarkers and Imaging of Breast Cancer. AJR Am J Roentgenol. 2018;210(2):271–8. 29166151 10.2214/AJR.17.18708

[61] Lee SH, Ryu HS, Jang MJ, Yi A, Ha SM, Kim SY, Glandular Tissue Component and Breast Cancer Risk in Mammographically Dense Breasts at Screening Breast US. Radiology. 2021;301(1):57–65. 34282967 10.1148/radiol.2021210367

[62] McCormack VA, dos Santos Silva I. Breast density and parenchymal patterns as markers of breast cancer risk: a meta-analysis. Cancer Epidemiol Biomarkers Prev. 2006;15(6):1159–69. 16775176 10.1158/1055-9965.EPI-06-0034

[63] Pettersson A, Graff RE, Ursin G, Santos Silva ID, McCormack V, Baglietto L, Mammographic density phenotypes and risk of breast cancer: a meta-analysis. J Natl Cancer Inst. 2014;106(5). 10.1093/jnci/dju078 PMC4568991 24816206

[64] Nassar A, Hoskin TL, Stallings-Mann ML, Degnim AC, Radisky DC, Frost MH, Ki-67 expression in sclerosing adenosis and adjacent normal breast terminal ductal lobular units: a nested case-control study from the Mayo Benign Breast Disease Cohort. Breast Cancer Res Treat. 2015;151(1):89–97. 25863475 10.1007/s10549-015-3370-y PMC4409576

[65] Huh SJ, Oh H, Peterson MA, Almendro V, Hu R, Bowden M, The Proliferative Activity of Mammary Epithelial Cells in Normal Tissue Predicts Breast Cancer Risk in Premenopausal Women. Cancer Res. 2016;76(7):1926–34. 26941287 10.1158/0008-5472.CAN-15-1927 PMC4873436

[66] Oh H, Eliassen AH, Wang M, Smith-Warner SA, Beck AH, Schnitt SJ, Expression of estrogen receptor, progesterone receptor, and Ki67 in normal breast tissue in relation to subsequent risk of breast cancer. NPJ Breast Cancer. 2016;2:16032-. 28111631 10.1038/npjbcancer.2016.32 PMC5243126

[67] Weinstat-Saslow D, Merino MJ, Manrow RE, Lawrence JA, Bluth RF, Wittenbel KD, Overexpression of cyclin D mRNA distinguishes invasive and in situ breast carcinomas from non-malignant lesions. Nat Med. 1995;1(12):1257–60. 7489405 10.1038/nm1295-1257

[68] Shoker BS, Jarvis C, Clarke RB, Anderson E, Hewlett J, Davies MP, Estrogen receptor-positive proliferating cells in the normal and precancerous breast. Am J Pathol. 1999;155(6):1811–5. 10595909 10.1016/S0002-9440(10)65498-3 PMC1866935

[69] Tucker H, Parsons C, Ellis S, Rhoads M, Akers R. Tamoxifen impairs prepubertal mammary development and alters expression of estrogen receptor α (ESR1) and progesterone receptors (PGR). Domestic animal endocrinology. 2016;54:95–105. 26619291 10.1016/j.domaniend.2015.10.002

[70] Russo J, Ao X, Grill C, Russo I. Pattern of distribution of cells positive for estrogen receptor α and progesterone receptor in relation to proliferating cells in the mammary gland. Breast cancer research and treatment. 1999;53:217–27. 10369068 10.1023/a:1006186719322

[71] Guo W, Keckesova Z, Donaher JL, Shibue T, Tischler V, Reinhardt F, Slug and Sox9 cooperatively determine the mammary stem cell state. Cell. 2012;148(5):1015–28. 22385965 10.1016/j.cell.2012.02.008 PMC3305806

[72] Parsa S, Ramasamy SK, De Langhe S, Gupte VV, Haigh JJ, Medina D, Terminal end bud maintenance in mammary gland is dependent upon FGFR2b signaling. Developmental biology. 2008;317(1):121–31. 18381212 10.1016/j.ydbio.2008.02.014

[73] Pond AC, Bin X, Batts T, Roarty K, Hilsenbeck S, Rosen JM. Fibroblast growth factor receptor signaling is essential for normal mammary gland development and stem cell function. Stem Cells. 2013;31(1):178–89. 23097355 10.1002/stem.1266 PMC3690809

[74] Muraoka-Cook RS, Sandahl MA, Strunk KE, Miraglia LC, Husted C, Hunter DM, ErbB4 splice variants Cyt1 and Cyt2 differ by 16 amino acids and exert opposing effects on the mammary epithelium in vivo. Molecular and cellular biology. 2009. 10.1128/MCB.01705-08 PMC2738276 19596786

[75] Long W, Wagner K-U, Lloyd KK, Binart N, Shillingford JM, Hennighausen L, Impaired differentiation and lactational failure of Erbb4-deficient mammary glands identify ERBB4 as an obligate mediator of STAT5. 2003. 10.1242/dev.00715 12954715

[76] Jones FE, Welte T, Fu X-Y, Stern DF. ErbB4 signaling in the mammary gland is required for lobuloalveolar development and Stat5 activation during lactation. The Journal of cell biology. 1999;147(1):77–88. 10508857 10.1083/jcb.147.1.77 PMC2164978

[77] Zhu Y, Sullivan LL, Nair SS, Williams CC, Pandey AK, Marrero L, Coregulation of estrogen receptor by ERBB4/HER4 establishes a growth-promoting autocrine signal in breast tumor cells. Cancer research. 2006;66(16):7991–8. 16912174 10.1158/0008-5472.CAN-05-4397

[78] Accornero P, Miretti S, Cucuzza LS, Martignani E, Baratta M. Epidermal growth factor and hepatocyte growth factor cooperate to enhance cell proliferation, scatter, and invasion in murine mammary epithelial cells. Journal of molecular endocrinology. 2010;44(2):115–25. 19850646 10.1677/JME-09-0035

[79] Garner OB, Bush KT, Nigam KB, Yamaguchi Y, Xu D, Esko JD, Stage-dependent regulation of mammary ductal branching by heparan sulfate and HGF-cMet signaling. Developmental biology. 2011;355(2):394–403. 21586278 10.1016/j.ydbio.2011.04.035 PMC3118867

[80] Murphy N, Knuppel A, Papadimitriou N, Martin RM, Tsilidis K, Smith-Byrne K, Insulin-like growth factor-1, insulin-like growth factor-binding protein-3, and breast cancer risk: observational and Mendelian randomization analyses with∼ 430 000 women. Annals of Oncology. 2020;31(5):641–9. 32169310 10.1016/j.annonc.2020.01.066 PMC7221341

[81] Martínez-Reza I, Díaz L, García-Becerra R. Preclinical and clinical aspects of TNF-α and its receptors TNFR1 and TNFR2 in breast cancer. Journal of Biomedical Science. 2017;24(1):1–8. 29202842 10.1186/s12929-017-0398-9 PMC5713022

[82] Dükel M, Streitfeld WS, Tang TCC, Backman LR, Ai L, May WS, The breast cancer tumor suppressor TRIM29 is expressed via ATM-dependent signaling in response to hypoxia. Journal of Biological Chemistry. 2016;291(41):21541–52. 27535224 10.1074/jbc.M116.730960 PMC5076825

[83] Liu J, Welm B, Boucher KM, Ebbert MT, Bernard PS. TRIM29 functions as a tumor suppressor in nontumorigenic breast cells and invasive ER+ breast cancer. The American journal of pathology. 2012;180(2):839–47. 22138580 10.1016/j.ajpath.2011.10.020 PMC5691328

[84] Shams A. Re-evaluation of the myoepithelial cells roles in the breast cancer progression. Cancer Cell International. 2022;22(1):1–16. 36510219 10.1186/s12935-022-02829-y PMC9746125

[85] Lengerke C, Fehm T, Kurth R, Neubauer H, Scheble V, Müller F, Expression of the embryonic stem cell marker SOX2 in early-stage breast carcinoma. BMC cancer. 2011;11:1–10. 21276239 10.1186/1471-2407-11-42 PMC3038979

[86] Chaturvedi S, Biswas M, Sadhukhan S, Sonawane A. Role of EGFR and FASN in breast cancer progression. Journal of Cell Communication and Signaling. 2023:1–34. 37490191 10.1007/s12079-023-00771-w PMC10713975

[87] Slaughter DP, Southwick HW, Smejkal W. Field cancerization in oral stratified squamous epithelium; clinical implications of multicentric origin. Cancer. 1953;6(5):963–8. 13094644 10.1002/1097-0142(195309)6:5<963::aid-cncr2820060515>3.0.co;2-q

[88] Young CD, Pfefferle AD, Owens P, Kuba MG, Rexer BN, Balko JM, Conditional loss of ErbB3 delays mammary gland hyperplasia induced by mutant PIK3CA without affecting mammary tumor latency, gene expression, or signaling. Cancer research. 2013;73(13):4075–85. 23633485 10.1158/0008-5472.CAN-12-4579 PMC3702683

[89] Liu R, Shi P, Zhou Z, Zhang H, Li W, Zhang H, Krüpple-like factor 5 is essential for mammary gland development and tumorigenesis. The Journal of pathology. 2018;246(4):497–507. 30101462 10.1002/path.5153

[90] Sumbal J, Koledova Z. FGF signaling in mammary gland fibroblasts regulates multiple fibroblast functions and mammary epithelial morphogenesis. Development. 2019;146(23):dev185306. 31699800 10.1242/dev.185306

[91] Mertelmeyer S, Weider M, Baroti T, Reiprich S, Fröb F, Stolt CC, The transcription factor Sox10 is an essential determinant of branching morphogenesis and involution in the mouse mammary gland. Scientific reports. 2020;10(1):17807. 33082503 10.1038/s41598-020-74664-y PMC7575560

[92] Satoh K, Hovey R, Malewski T, Warri A, Goldhar A, Ginsburg E, Progesterone enhances branching morphogenesis in the mouse mammary gland by increased expression of Msx2. Oncogene. 2007;26(54):7526–34. 17546050 10.1038/sj.onc.1210555

[93] Heaphy CM, Griffith JK, Bisoffi M. Mammary field cancerization: molecular evidence and clinical importance. Breast Cancer Res Treat. 2009;118(2):229–39. 19685287 10.1007/s10549-009-0504-0

[94] Curtius K, Wright NA, Graham TA. An evolutionary perspective on field cancerization. Nature Reviews Cancer. 2018;18(1):19–32. 29217838 10.1038/nrc.2017.102

[95] Gendzekhadze K, Gaidulis L, Senitzer D. Chimerism testing by quantitative PCR using Indel markers. Methods Mol Biol. 2013;1034:221–37. 23775739 10.1007/978-1-62703-493-7_11

[96] Vicente DC, Laranjeira AB, Miranda EC, Yunes JA, de Souza CA. Chimerism interpretation with a highly sensitive quantitative PCR method: 6 months median latency before chimerism drop below 0.1. Bone Marrow Transplant. 2016;51(6):874–5. 26878657 10.1038/bmt.2016.5

[97] Luche H, Weber O, Nageswara Rao T, Blum C, Fehling HJ. Faithful activation of an extra-bright red fluorescent protein in “knock-in” Cre-reporter mice ideally suited for lineage tracing studies. Eur J Immunol. 2007;37(1):43–53. 17171761 10.1002/eji.200636745

[98] Lim E, Vaillant F, Wu D, Forrest NC, Pal B, Hart AH, Aberrant luminal progenitors as the candidate target population for basal tumor development in BRCA1 mutation carriers. Nat Med. 2009;15(8):907–13. 19648928 10.1038/nm.2000

[99] Molyneux G, Geyer FC, Magnay FA, McCarthy A, Kendrick H, Natrajan R, BRCA1 basal-like breast cancers originate from luminal epithelial progenitors and not from basal stem cells. Cell Stem Cell. 2010;7(3):403–17. 20804975 10.1016/j.stem.2010.07.010

[100] Bolíbar B, Avilés FF, Morros R, del Mar Garcia-Gil M, Hermosilla E, Ramos R, SIDIAP database: electronic clinical records in primary care as a source of information for epidemiologic research. Medicina clinica. 2012;138(14):617–21. 22444996 10.1016/j.medcli.2012.01.020

[101] Recalde M, Rodríguez C, Burn E, Far M, García D, Carrere-Molina J, Data resource profile: the information system for research in primary care (SIDIAP). International Journal of Epidemiology. 2022;51(6):e324–e36. 35415748 10.1093/ije/dyac068 PMC9749711

[102] Marti A, Jehn B, Costello E, Keon N, Ke G, Martin F, Protein kinase A and AP-1 (c-Fos/JunD) are induced during apoptosis of mouse mammary epithelial cells. Oncogene. 1994;9(4):1213–23. 8134124

[103] Temko D, Cheng Y-K, Polyak K, Michor F. Mathematical modeling links pregnancy-associated changes and breast cancer risk. Cancer research. 2017;77(11):2800–9. 28360138 10.1158/0008-5472.CAN-16-2504 PMC5477484

[104] Strange R, Metcalfe T, Thackray L, Dang M. Apoptosis in normal and neoplastic mammary gland development. Microscopy research and technique. 2001;52(2):171–81. 11169865 10.1002/1097-0029(20010115)52:2<171::AID-JEMT1003>3.0.CO;2-T

[105] Haricharan S, Dong J, Hein S, Reddy JP, Du Z, Toneff M, Mechanism and preclinical prevention of increased breast cancer risk caused by pregnancy. Elife. 2013;2:e00996. 24381245 10.7554/eLife.00996 PMC3874103

[106] Martinson HA, Jindal S, Durand-Rougely C, Borges VF, Schedin P. Wound healing-like immune program facilitates postpartum mammary gland involution and tumor progression. International Journal of Cancer. 2015;136(8):1803–13. 25187059 10.1002/ijc.29181 PMC4324053

[107] Anders CK, Hsu DS, Broadwater G, Acharya CR, Foekens JA, Zhang Y, Young age at diagnosis correlates with worse prognosis and defines a subset of breast cancers with shared patterns of gene expression. Journal of clinical oncology. 2008;26(20):3324–30. 18612148 10.1200/JCO.2007.14.2471

[108] Liede A, Mansfield CA, Metcalfe KA, Price MA, Cancer KCFCfRiF B, Snyder C, Preferences for breast cancer risk reduction among BRCA1/BRCA2 mutation carriers: a discrete-choice experiment. Breast cancer research and treatment. 2017;165:433–44. 28624978 10.1007/s10549-017-4332-3 PMC5543193

[109] Rains CP, Bryson HM, Fitton A. Cabergoline: a review of its pharmacological properties and therapeutic potential in the treatment of hyperprolactinaemia and inhibition of lactation. Drugs. 1995;49:255–79. 7729332 10.2165/00003495-199549020-00009

[110] Nair AB, Jacob S. A simple practice guide for dose conversion between animals and human. Journal of basic and clinical pharmacy. 2016;7(2):27. 27057123 10.4103/0976-0105.177703 PMC4804402

[111] Smalley MJ. Isolation, culture and analysis of mouse mammary epithelial cells. Mouse Cell Culture: Methods and Protocols. 2010:139–70. 10.1007/978-1-59745-019-5_11 20204626

[112] Prieto C, Barrios D. RaNA-Seq: Interactive RNA-Seq analysis from FASTQ files to functional analysis. Oxford University Press; 2020. 10.1093/bioinformatics/btz854 31730197

[113] Patro R, Duggal G, Love MI, Irizarry RA, Kingsford C. Salmon provides fast and bias-aware quantification of transcript expression. Nature methods. 2017;14(4):417–9. 28263959 10.1038/nmeth.4197 PMC5600148

[114] Robinson MD, McCarthy DJ, Smyth GK. edgeR: a Bioconductor package for differential expression analysis of digital gene expression data. bioinformatics. 2010;26(1):139–40. 19910308 10.1093/bioinformatics/btp616 PMC2796818

[115] Wu T, Hu E, Xu S, Chen M, Guo P, Dai Z, clusterProfiler 4.0: A universal enrichment tool for interpreting omics data. The innovation. 2021;2(3). 10.1016/j.xinn.2021.100141 PMC8454663 34557778

[116] Yu G, Wang L-G, Han Y, He Q-Y. clusterProfiler: an R package for comparing biological themes among gene clusters. Omics: a journal of integrative biology. 2012;16(5):284–7. 22455463 10.1089/omi.2011.0118 PMC3339379

[117] Husby A, Wohlfahrt J, Øyen N, Melbye M. Pregnancy duration and breast cancer risk. Nature communications. 2018;9(1):4255. 10.1038/s41467-018-06748-3 PMC6199327 30353005

